# Structural Mechanism of an Efficacy Photoswitch Targeting the β_2_‐adrenergic Receptor

**DOI:** 10.1002/anie.202517995

**Published:** 2026-03-18

**Authors:** Robin Stipp, Quentin Bertrand, Matilde Trabuco, Anna Duran‐Corbera, Maria Tindara Ignazzitto, Hannah Glover, Fabienne Stierli, Juanlo Catena, Melissa Carrillo, Sina Hartmann, Hans‐Peter Seidel, Matthias Mulder, Thomas Mason, Yasushi Kondo, Maximillian Wranik, Martin Appleby, Christoph Sager, Raymond Sierra, Gregory Gate, Pamela Schleissner, Xinxin Cheng, Tobias Weinert, Robert Cheng, Sandra Mous, John H. Beale, Michal Kepa, Amadeu Llebaria, Michael Hennig, Xavier Rovira, Joerg Standfuss

**Affiliations:** ^1^ PSI Center for Life Sciences, Paul Scherrer Institute Villigen Switzerland; ^2^ LeadXpro AG Villigen Switzerland; ^3^ MCS, Laboratory of Medicinal Chemistry Institute For Advanced Chemistry of Catalonia (IQAC), CSIC Barcelona Spain; ^4^ Synthesis of High Added Value Molecules (SIMChem) Institut De Química Avançada De Catalunya (IQAC‐CSIC) Barcelona Spain; ^5^ Stanford Cancer Institute Stanford University School of Medicine Stanford California USA; ^6^ PSI Center for Photon Science Paul Scherrer Institute Villigen Switzerland; ^7^ Linac Coherent Light Source SLAC National Accelerator Laboratory Menlo Park California USA; ^8^ Department of Chemistry University of Zurich Zurich Switzerland

**Keywords:** activity modulation, azobenzene photoswitches, G protein‐coupled receptors, photopharmacology, time‐resolved serial femtosecond crystallography

## Abstract

The field of photopharmacology develops light‐responsive drugs that can modulate protein activity, enabling precise and dynamic investigations of their roles in health and disease. Adrenergic receptors are prominent targets for this approach because they are prototypical G protein‐coupled receptors with high clinical relevance in bronchial and cardiovascular diseases. Here, we employed the azobenzene‐based compound photoazolol‐1 in combination with time‐resolved serial crystallography at X‐ray free‐electron lasers to resolve the molecular mechanisms by which photoswitchable β‐blockers modulate activity of the β_2_‐adrenoceptor (β_2_AR). Time‐resolved structures of the receptor bound to *trans*‐photoazolol‐1 (pre‐photoconversion), a strained intermediate in the nanosecond range, and the fully photoisomerized *cis*‐photoazolol‐1 reveal how isomerization of the azobenzene moiety induces distinct conformational changes within the orthosteric ligand binding pocket. Within seconds, light‐excited photoazolol‐1 adopts a new binding pose, altering interactions with extracellular loop 2 and shifting the positions of transmembrane helices 5, 6, and 7. Functional assays of β_2_AR in cellular membranes show that photoazolol‐1 acts as an efficacy photoswitch, changing from an inverse agonist to a neutral antagonist upon isomerization without leaving the binding pocket. In combination, these findings suggest a molecular mechanism for activity modulation via efficacy photoswitches and provide a framework for designing ligands that exploit light‐driven transitions within the binding pocket to achieve spatiotemporal control of receptor function.

## Graphical abstract

Time‐resolved structures from x‐ray free‐electron laser measurements combined with cellular assays demonstrate how the photoswitchable β‐blocker photoazolol‐1 acts as an efficacy switch of β_2_‐adrenergic receptor function. Light‐induced ligand repositioning shifts activity from inverse agonism toward neutral antagonism by altering local helical interactions. These findings provide a structural roadmap for designing ligands with improved optical control of receptor function.

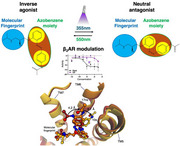

## 1 Introduction

G protein‐coupled receptors (GPCRs) are central to many human signaling pathways and are common targets in drug development [1]. In cases where disease disrupts natural homeostasis, many GPCRs serve as essential “adjustment knobs” to restore physiological equilibrium. β‐adrenergic receptors are prominent GPCRs that regulate the body's physiological responses to stress. Small‐molecule drugs like propranolol, known as β‐blockers, inhibit adrenergic receptor activity by blocking the binding site for endogenous adrenaline and noradrenaline to reduce heart rate and blood pressure in patients with different cardiovascular issues. The specificity of these drugs for adrenergic receptor subtypes, along with their action as agonists or antagonists, is critical for minimizing off‐target effects.

The β_2_‐adrenergic receptor (β_2_AR) acts as a reference to study the molecular pharmacology of GPCR activation [2] due to the early availability of both ligand‐bound [3, 4, 5, 6] and G protein‐bound states [4]. Some of the secrets behind the adaptations of the protein to different ligands have been uncovered using multiple methods, including time‐resolved cryo‐electron microscopy [7], nuclear magnetic resonance spectroscopy [8] and molecular dynamics simulations [9]. Experimentally resolving structures at atomistic resolution to capture these adaptations has great potential to advance our understanding of how ligands modulate receptor activity, with important implications for modern drug discovery [10].

Rhodopsin constitutes another important system to understand GPCR activation [10] as it can be purified from native sources and can be triggered efficiently by an ultrafast photoreaction that alters the bound retinal chromophore from an inverse agonist to a full agonist. Photoactive ligands like retinal are ideal for time‐resolved studies as they allow synchronized initiation of a specific process to be observed. However, they are not available for most GPCRs, prohibiting the direct study of ligand interaction dynamics on the structural level. Exceptions are created by the field of photopharmacology. The field focuses on chemically modifying ligands to put pharmacologically relevant targets under the control of light to achieve superior temporal and spatial control over target activity compared to conventional medications [11, 12, 13]. Azobenzene‐based compounds, with their modifiable chemical scaffolds and high quantum yields [14, 15], have driven recent advancements, including photoswitchable agonists [16] and antagonists [17, 18] targeting adrenergic receptors, as well as applications in zebrafish [19] and mice [20].

One such photoswitch is photoazolol‐1 (or PZL‐1), a compound derived from propranolol, the first major β–receptor–blocking drug to be used clinically [21]. Similarly to its parent compound, photoazolol‐1 has a high affinity and acts as a potent β‐blocker based on cell‐based cAMP reporter assays [18]. It retains the characteristic fingerprint region common to many synthetic β_2_AR ligands and the endogenous adrenaline and noradrenaline hormones but replaces the aromatic moiety, present in related β_2_AR antagonists, with a *p*‐acetamido substituted azobenzene to allow for efficient switches between *trans* and *cis* configurations [18]. In its metastable *cis*‐form, photoazolol‐1 has been shown to have a considerably lower antagonistic potency than the *trans*‐isomer [18]; however, whether this effect is solely based on a loss of affinity or a change in receptor‐ligand interaction dynamics is not understood.

Time‐resolved femtosecond serial crystallography (TR‐SFX) [22, 23] allows structural snapshots to be resolved at X‐ray Free Electron Lasers (XFELs) over a wide temporal range down to femtoseconds but has also been adapted to resolve slower intermediates at synchrotron sources [24, 25]. The technique has been employed on a growing series of proteins [25, 26] and has been particularly successful with light‐triggered proteins, including rhodopsin, the GPCR central to our visual system [27]. For the study of other pharmacologically relevant GPCRs, similarly efficient trigger systems are needed.

In this study, we demonstrate the use of photoazolol‐1 to resolve receptor‐ligand interaction dynamics in the β_2_AR at XFELs. We determined the room‐temperature structure of the photoazolol‐1/β_2_AR complex at 2.5 Å resolution and investigated the effect of illuminating photoazolol‐1 within its binding pocket using TR‐SFX at the Linac Coherent Light Source (LCLS) and the Swiss X‐ray Free Electron Laser (SwissFEL). Photoisomerization repositions the ligand within nanoseconds, but counter to our initial expectations, even after several seconds, photoazolol‐1 does not leave the receptor. Instead, its *p*‐acetamido azobenzene group finds a new position between transmembrane helix 5 and 6 (TM5 and TM6), where it disrupts the packing of the seven transmembrane helical bundle and induces adaptive conformational changes in nearby residues related to receptor activation. Cellular assays, together with comparisons of these rearrangements to early changes in the photoactivation of the native retinal chromophore in visual rhodopsin, suggest that *trans‐*photoazolol‐1 has inverse agonist activity that is abolished upon illumination, offering new insights into receptor modulation and the molecular basis of photopharmacology.

## 2 Results and Discussion

### 2.1 Photoazolol‐1 Binding Mode

**FIGURE 1:**
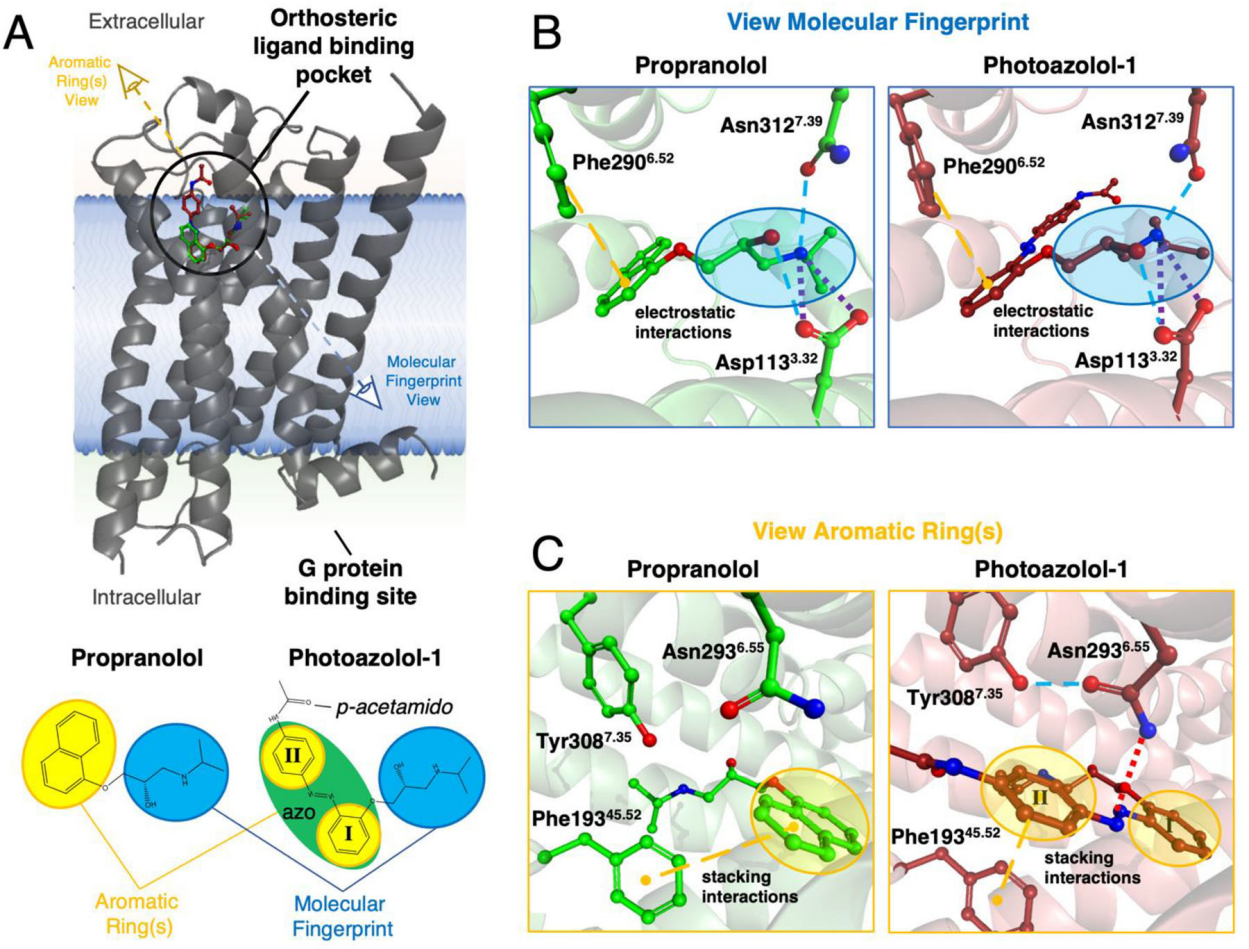
Comparison of propranolol and photoazolol‐1 binding. (A) The β_2_AR follows the prototypical seven‐transmembrane (7TM) fold (grey cartoon) of GPCRs with the orthosteric ligand binding site (propranolol (green) and photoazolol‐1 (red) shown in ball and sticks representation) close to the extracellular and the G protein‐binding site on the intracellular side of the membrane. The chemical structures of propanolol and photoazolol‐1 are similar, sharing the molecular fingerprint region (blue) and the aromatic rings (yellow) which are connected by an ‐N = N‐ linkage to form an azobenene motif (green). (B) Comparison of the structures of the propranolol‐bound (pdb code: 6ps5) [30] and photoazolol‐1‐bound receptor illustrates how the molecular fingerprint region binds the β_2_AR. The ligand binds to the receptor through ionic interactions (purple dotted lines) and hydrogen bonds (blue dashed lines). This binding is supported by hydrophobic stacking interactions (yellow dashed lines) at the adjacent aromatic ring. Oxygen atoms are shown in red and nitrogen atoms in blue. (C) Photoazolol‐1 retains the general chemical scaffold of propranolol but separates the two aromatic rings via a photoswitchable azobenzene group (green) and introduces an acetamido group on the para position of ring II. The key interactions of the original β‐blocker with the β_2_AR binding pocket are preserved despite the modifications in this synthetic photoswitch.

One key challenge in photopharmacology is designing photoswitchable ligands that retain the binding mode of the parent compounds in one of their configurations. To compare the binding mode of *trans*‐photoazolol‐1 to conventional β‐blockers, we first solved the crystal structure of β_2_AR bound to dark‐equilibrated photoazolol‐1 (Figure 1A). Notably, even after thorough optimization our crystals diffracted only to low resolution using conventional cryo‐crystallography at synchrotrons, as is not uncommon for GPCR microcrystals [28]. High‐intensity XFEL pulses were essential to reach resolutions of 2.5 Å (Table S1), which are typically required to accurately model ligand and water‐mediated interactions for structure‐guided drug design applications. The inability to grow large, well‐diffracting crystals may be due to the chosen construct, which contains a T4 lysozyme fusion and terminal truncations to facilitate crystallization but only a single mutation (compare material and methods), as systematic thermostabilization approaches often have the drawback of limiting conformational flexibility of the targeted receptor [29]. A root‐mean‐square deviation of Cα atoms of 0.638Å to the propranolol‐bound β_2_AR structure from LCLS [30] shows the photoazolol‐1‐bound structure is near identical, confirming the successful design of the photoswitchable compound. The high degree of similarity includes the fingerprint region (Figure 1B) of photoazolol‐1, which interacts with the receptor binding pocket through the same interactions as propranolol, including the characteristic polar contacts with Asp113^3.32^ and Asn312^7.38^ [superscripts denote Ballesteros‐Weinstein numbers [31]], similarly found in other adrenoceptor‐ligand complexes [32, 33] and known to be critical for potency and efficacy of agonist activation [34, 35].

The photoswitchable azobenzene moiety maintains π–π interactions with Phe193^45.52^ and Phe290^6.52^, despite a doubling in the distance between the aromatic rings in photoazolol‐1 compared to propranolol (Figure 1C). A difference in the binding mode of *trans‐*photoazolol‐1 compared with propranolol, is the additional polar interaction between the azobenzene bond of the former molecule and Asn293^6.55^, which changes the rotamer to establish this contact. This interaction is further stabilized by an additional hydrogen bond between Asn293^6.55^ and Tyr308^7.35^. Interestingly, this tyrosine is replaced by a phenylalanine in the β_1_‐adrenergic receptor, suggesting a degree of subtype specificity as observed for the related opto‐prop‐1 molecule [17]. The overall similar binding mode of *trans*‐photoazolol‐1 (Figure S1) is reflected in a comparable binding affinity: 9.8 nM for propranolol and 14.1 nM for dark‐adapted photoazolol‐1 (Figure S2). The design of photoazolol‐1 thus resulted in a compound where both binding mode and binding affinity are similar to other known β‐blockers, fulfilling one of the key requirements for an efficient photochemical trigger of β_2_AR activity.

### 2.2 Structural Effects of Photoazolol‐1 Illumination

Another key requirement for an effective photopharmacological compound is its ability to induce a change in its biological effect upon illumination. Photoazolol‐1 converts efficiently in various buffers and an excess of β_2_AR only marginally slows the reaction suggesting that the binding pocket does not limit photoisomerization (Figure S3). Irradiation furthermore results in a 17‐fold reduction in antagonistic activity, even though only 86% of the compound converts to *cis*‐photoazolol‐1 in the photostationary state [18]. To investigate the molecular basis of this effect, we performed time‐resolved serial crystallography experiments during two beamtimes at LCLS and SwissFEL. At LCLS, we employed an injector‐based approach to probe an early time delay of 17 ns, twice the pulse length of the used optical laser. Based on previous experience with retinal proteins [27, 36, 37] and the ultrafast kinetics of azobenzene isomerization [38, 39], we expected to resolve the initial effect of photoazolol‐1 isomerization on the receptor. Such a classical TR‐SFX approach is, however, unsuitable for longer time delays as it is limited by extrusion stability and the achievable distance between pump‐probe pulses. At SwissFEL, we therefore relied on a fixed‐target approach to resolve the effects on the receptor after approximately 10 s of photoactivation (hereafter called 10 s). The differences and similarities between the two experiments are further explained in Figure S4. Progressing from the starting point of the *trans*‐photoazolol‐1 structure, isomorphous difference electron density maps [*F*_o_(light) − *F*_o_(dark)] enabled us to track changes in protein‐ligand interactions (Figure S5) over time. To further analyze these structural changes, we determined extrapolated structure factors using an activation level of 28% for the 17 ns and 15% for the 10 s time delays. While we cannot exclude an ensemble of structures with lower occupancies, single molecular structures refined well against the extrapolated data. The potential impact of data extrapolation could be further limited by combining the dark and light models and refining them against the overall data at each time delay. A morph between the structure highlights the conformational changes in the binding pocket and along 7TM helical bundle (Movie S1).

**FIGURE 2:**
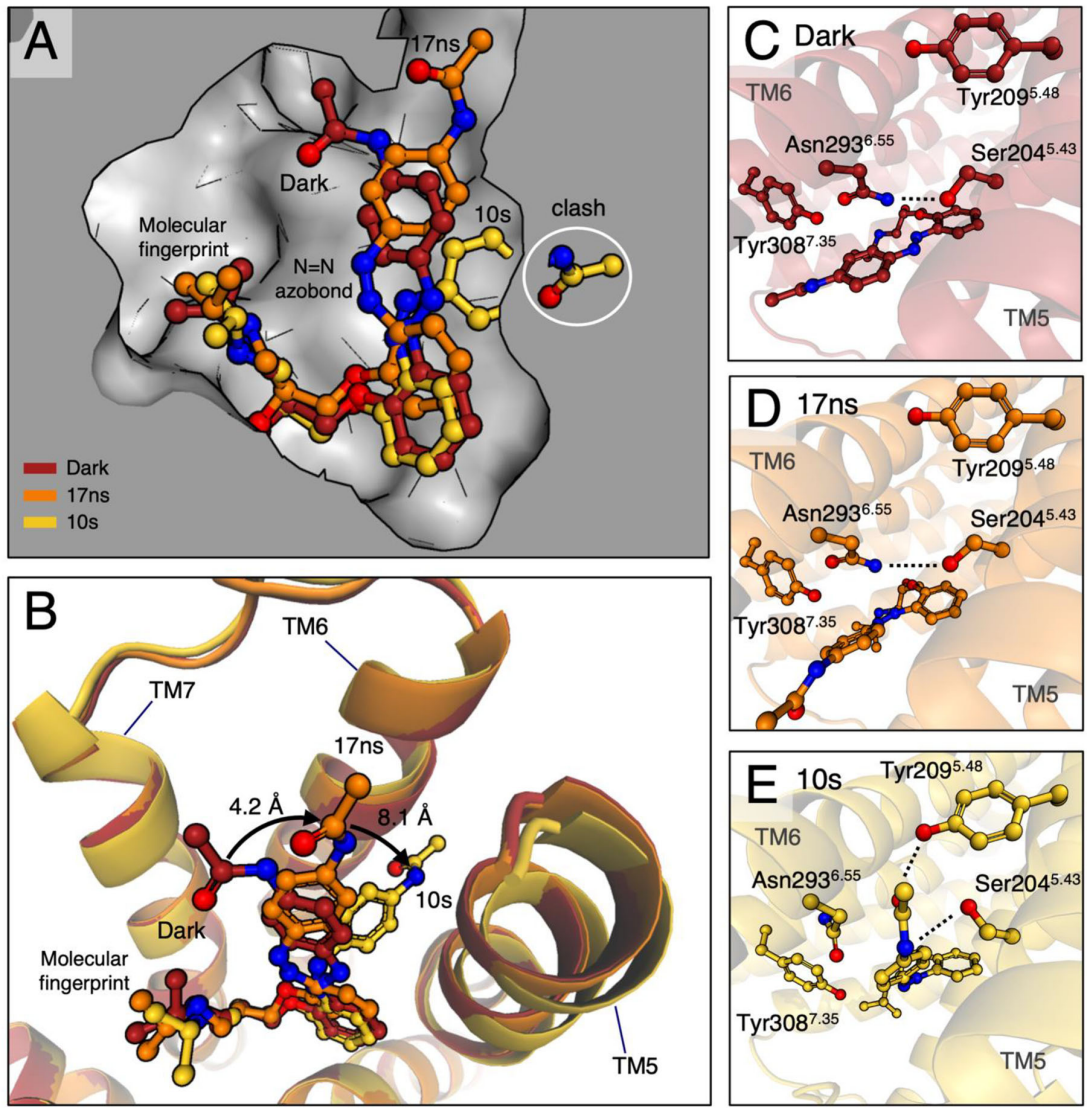
Structural effects of photoazolol‐1 isomerization. (A) The binding pocket of the β_2_AR dark state (grey) with superposed photoazolol‐1 (dark state in red, 17 ns in orange and 10 s in gold). The initially twisted *cis*‐photoazolol‐1 fits the binding pocket, while relaxation at the later time delay would lead to a clash with the interface between TM5 and TM6. (B) Photoazolol‐1 isomerization leads to a relocation (black arrow) of the *p*‐acetamido substitutions into a new position between TM5 and TM6. Superposition of the dark (red), 17 ns (orange), and 10 s structures (gold) show how the binding pocket adapts through an outward move of TM5 and TM6 combined with an inward shift of TM7. Oxygen atoms are shown in red and nitrogen atoms in blue. In the dark (C) and after 17 ns (D), the ionic interactions (dotted lines) between Ser204^5.43^ and Asn293^6.55^ are maintained, while full photoazolol‐1 isomerization after about 10 s (E) interrupts this interaction implicated in agonist‐induced β_2_AR activation.

The structures show that photoazolol‐1 adopts a strained *cis* configuration 17 ns after illumination, leading to only minimal changes in the receptor (Figure 2). Unable to chemically relax within the tight constraints of the binding pocket, the photoswitch maintains much of its original shape. The light‐induced isomerization, however, causes a shift in the *p*‐acetamido azobenzene moiety by 4.2 Å (measured between the respective positions of the CO2 atom), apparently weakening interactions with the receptor. Specifically, the π–π interactions with Phe290^6.52^ are lost, and increased distances with less favorable geometry suggest weakened interactions with Phe193^45.52^ and Asn293^6.55^. Despite these alterations, the ligand remains anchored through strong polar interactions mediated by the fingerprint region. Given the size limitations of the binding pocket and the minimal changes in the receptor during this early time delay, it seems likely that the initial photoreaction proceeds without a substantial rotational component around the azo bond. These early rearrangements leave the ligand in a metastable pose with its stored strain energy preserved to induce conformational changes in the protein – providing an observation window into the dynamic response of a GPCR to its bound ligand – similar to the response of rhodopsin after initial photoisomerization before the receptor adapts the active conformation [27].

In our previous studies investigating protein‐ligand interaction dynamics in tubulin [40, 41], the adenosine A2a receptor [42] and the metabotropic glutamate receptor 5 [43], the synthetic photoswitches left the binding pocket within milliseconds after activation. In contrast, photoazolol‐1 remains bound to the β_2_AR even after 10 s, three orders of magnitude longer than we could probe in our previous experiments. In contrast, within 10 s after illumination the initially strained *cis*‐photoazolol‐1 configuration relaxed into a more favorable geometry (Figure 2) with slightly higher total energy than the original *trans*‐state (Figure S1D). These energetic considerations are consistent with ligand retention in the binding pocket several seconds after isomerization. After relaxation of the transient photoazolol‐1 configuration, the *p*‐acetamido group, measured between the two CO2 labelled atoms, has shifted further with a maximum distance of 8.1 Å. The group finds a new position between TM5 and TM6, where it leads to a rotamer change of Ser204^5.43^ and Asn293^6.55^, interrupting the interaction between these two residues and establishing new interactions with Tyr209^5.48^ and Ser204^5.43^. The repositioned ligand has shifted the ends of TM5 and TM6 away from the original binding pocket, whereas TM7 including Tyr308^7.35^ (Figure S6) moves in on the other side, keeping the overall volume of the binding pocket largely the same. Comparisons between structures containing ligands with diverse pharmacological profiles have implicated similar interactions with residues in TM5 and TM6 in agonist‐induced activation of β‐adrenergic receptors [32, 44, 45, 46]. However, despite these local rearrangements within the ligand‐binding pocket, general structural hallmarks of GPCR activation (including the DR^3.50^Y, CW^6.48^xP, and NP^7.50^xxY motifs) remain in an inactive conformation. High‐resolution structural studies have shown that ligand binding alone typically does not stabilize the fully active conformation and, instead, coupling to a signaling protein or mimetic is required to capture the active state in structural experiments [5, 47]. Accordingly, photoazolol‐1 isomerization alters local interactions within the orthosteric binding pocket associated with receptor activation but does not propagate into large‐scale conformational changes on the intracellular side where the G protein binds, consistent with the observed shift from inverse agonism to neutral antagonism upon illumination (see Discussion below).

### 2.3 Molecular Mechanism of Photoazolol‐1 Activity

**FIGURE 3:**
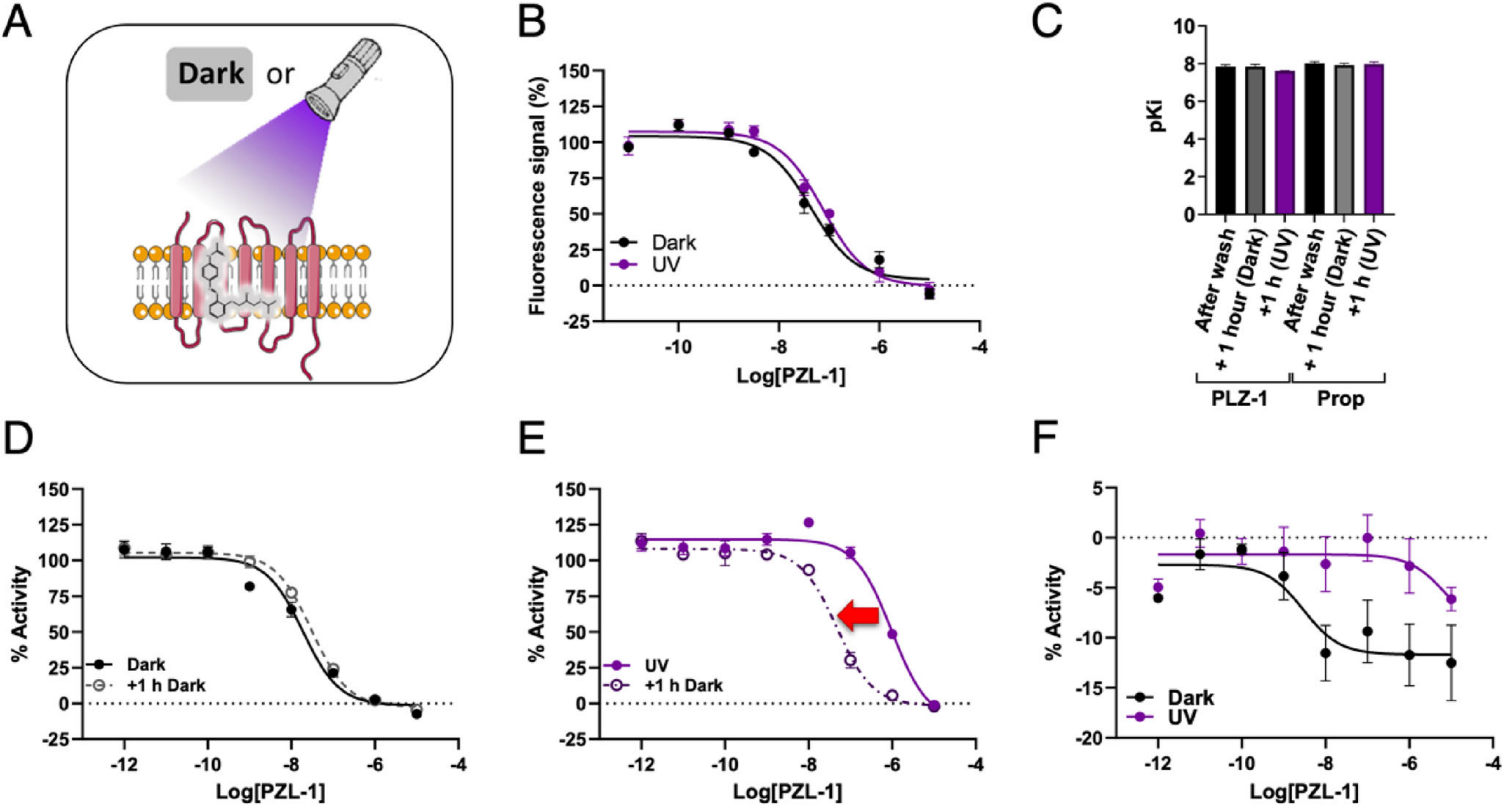
Light‐dependent binding and activity of photoazolol‐1 (PZL‐1) on the β_2_AR. (A) Schematic representation of the pharmacological protocol in which cell cultures were either kept in the dark or exposed to light for 15 min following a 1‐hour incubation with ligands. A thorough wash was then performed to remove any residual ligand from the medium. (B) Competitive binding curves of photoazolol‐1 with a constant concentration of the fluorescent ligand carazolol‐KK114 (100 nM). Measurements were performed 1 h after washing. (C) Plot of the negative logarithm of the inhibition constant (pKI) values as calculated from the competitive binding curves. (D) Dose‐response curves of photoazolol‐1 with a constant concentration of the agonist cimaterol (10 nM) 15 min after washing kept in the dark (solid black lines), and after an additional 1‐hour incubation in the dark (dashed black lines). (E) Dose‐response curves of photoazolol‐1 with a constant concentration of the agonist cimaterol (10 nM) 15 min after washing kept under light at 380 nm (solid violet lines) and after an additional 1‐hour incubation in the dark (dashed violet lines). (F) Dose‐response curves of photoazolol‐1 in β_2_AR overexpressing transfected cells in the absence of agonist and 15 min after washing in the dark or under illumination with light at 380 nm. Data are shown as the mean ± standard error of the mean (SEM) of three independent experiments in duplicate.

A guiding principle of azobenzene‐based approaches to photopharmacology, is the design of small molecules with differential function between *cis* and *trans* configurations. Our time‐resolved observations shed light on this mechanism with photoazolol‐1, a ligand that remains in the binding pocket but exerts striking differential activity upon photoisomerization. To investigate this observation, we characterized its dark and light‐activated forms in the cellular context using binding competition assays based on the fluorescent ligand Carazolol‐KK114 (Car‐KK114) which competes with the azobenzene ligands for the same β_2_AR binding site [48] (Figure 3A). Our results reveal that photoazolol‐1 binding to the β_2_AR is stable, showing no evidence of unbinding under any of the tested conditions, confirming what was observed in the structures. Indeed, competitive assays suggest that photoazolol‐1 remains bound to the receptor for over an hour following a thorough washing protocol, regardless of whether the samples were kept in the dark or illuminated (Figures 3B, C and Figure S2A). These results are very similar to those found for propranolol (Figure S2B) and the previously described opto‐prop‐1, a chemically related molecule without the *p*‐acetamido group, which did not undergo significant UV‐induced unbinding either. In contrast, opto‐prop‐2, the meta‐substituted photoswitchable β‐blocker, demonstrated light‐dependent unbinding [17]. Although photoazolol‐1 remains in the receptor pocket, a right shift of the dose‐response curve is observed in functional assays when cells are irradiated with a 380 nm light, showing a significant 53‐fold change in potency (Figures 3D, E, and Table 1). This could indicate that the isomerization to the *cis* isomer results in a loss of photoazolol‐1 antagonist activity, even though the ligand remains bound. Of note, no change in potency is observed for the control antagonist propranolol upon illumination (Figure S7 and Table 1). Strikingly, this functional antagonism is restored one‐hour post‐illumination, without additional ligand application (Figure 3E), suggesting reversibility through thermal relaxation back to the *trans* isomer. Very similar results were obtained when competing with higher concentrations of cimaterol (1 µM) for both photoazolol‐1 and propranolol (Figure S8). Of note here is that previous studies demonstrated that photoazolol‐1 relaxes to the *trans* isomer more rapidly in functional assays on β_2_AR‐expressing cells than in aqueous solution in vitro [Figure S11 in Duran‐Corbera et al. 2020 [18]], thus supporting the hypothesis that it does not leave the binding site and that the *cis* form is less stable than the *trans* in the context of the receptor.

**TABLE 1:** Pharmacological data.^a^

	DARK		LIGHT			
Compound	pEC_50_	SEM	pEC_50_	SEM	PPS^a^	SEM
Photoazolol‐1	7,73	0,03	6,01^****^	0,04	52,81	4,16
Photoazolol‐1 (+ 1 h)	7,51	0,02	7,31	0,09	1,65	0,14
Propranolol	8,08	0,09	7,76	0,07	2,08	0,08
Propranolol (+ 1 h)	8,02	0,03	7,85	0,05	1,50	0,11

^a^ PPS refers to Photoinduced Potency Shift, which is the relation (fold shift) between the measured half maximal effective concentration (expressed as negative logarithm pEC_50_) in light and dark conditions. Standard errors of the mean (SEM) from three independent experiments measured in duplicate are given. Statistical differences from EC_50_ values between experiments measured right after and 1 h after illumination are denoted for adjusted p‐values as follows: ****p < 0.0001.

To demonstrate that photoazolol‐1 is a light‐sensitive antagonist, we performed cellular activation assays in absence of the agonist cimaterol using a FRET‐based biosensor of cAMP concentration [18, 49]. In these assays, no agonist activity of photoazolol‐1 was observed when applied to cells overexpressing the β_2_AR (Figure 3F and Figure S9). On the contrary, photoazolol‐1 decreased the basal activity, thus acting as inverse agonist in the dark. This effect was abolished upon illumination, indicating that the *cis* isomer acts as a neutral antagonist instead. This difference in functional activity between the *trans* and *cis* isomers is consistent with our structures, showing that the *cis* isomer adopts a distinct binding pose, altering key interactions within the binding pocket. Notably, the establishment of new contacts with Ser204^5.43^ and Tyr209^5.48^, and the loss of contact with Phe193^45.52^, a residue implicated in receptor activation and ligand efficacy, correlates with this differential activity (Figures 1C and 3E). Indeed, Phe193^45.52^ and adjacent residues have been highlighted in multiple studies as critical positions for ligand specificity and activity modulation [e.g., constrained catecholamines [50]; β_1_ vs. β_2_ selectivity [51]; salmeterol binding [46]]. A recent study based on azo variants of clenbuterol has reported a photoinduced efficacy shift from partial agonist (*trans*) to antagonist (*cis*) [52] and a recent characterization of photoswitchable covalent ligands [53] provide further evidence that β_2_AR activity can be modulated by changing interactions in response to light without leaving the binding pocket.

Possible explanations to reconcile the persistence of photoazolol‐1 in the binding pocket with the light‐dependent loss and recovery of functional activity could be the following. It is plausible that photoazolol‐1 remains in the orthosteric pocket while exposing or allowing access to a secondary binding site for agonists only when bound in its *cis* state. This could be due to the loss of interaction between *cis*‐photoazolol‐1 and Phe193^45.52^, a residue potentially involved in the pocket reorganization and the agonist coordination at a secondary site within the entrance vestibule located between the extracellular loop 2 (ECL2), TM5 and TM6. Importantly, many changes are observed in the residues located in this region between the structures in complex with the *trans‐* and *cis*‐photoazolol‐1. These include amino acid rotamer differences of His93^2.64^, His296^6.58^, Lys305^7.32^, Tyr308^7.35^, and Asp192^45.51^ (Figure S6). This secondary binding site has been described for norepinephrine [54]. In addition, the exosite is occupied by the aryloxyalkyl tail of salmeterol as observed in the β_2_AR co‐crystalized structure [46]. Interestingly, this site is also engaged by homobivalent bitopic ligands composed of two alprenolol‐based β‐blockers connected via a long linker [55]. Moreover, similar mechanisms have been described in other GPCRs for allosteric molecules binding at this location, such as muscarinic acetylcholine receptors [56]. Therefore, structural, binding and functional evidence support the hypothesis of the agonist binding into a second site at the same time as *cis*‐photoazolol‐1, a combination that would induce the activation of the receptor. An alternative explanation is that photoazolol‐1 temporarily dissociates from the receptor, but remains in a nonwashable, membrane‐adjacent compartment, from which it can rebind. This mechanism has been described for other ligands targeting the β_2_AR [57, 58]. Finally, we cannot rule out the possibility of the observed functional changes being driven by *cis*‐specific allosteric effects from interacting proteins or from the existence of distinct receptor populations that differentially respond to specific ligand isomers. However, these two last‐mentioned hypotheses would not fit with the time‐resolved crystallography structural data, which suggest a stable binding of photoazolol‐1 in both states within the orthosteric site of β_2_AR.

### 2.4 Comparison to Natively Light‐activated GPCR

**FIGURE 4:**
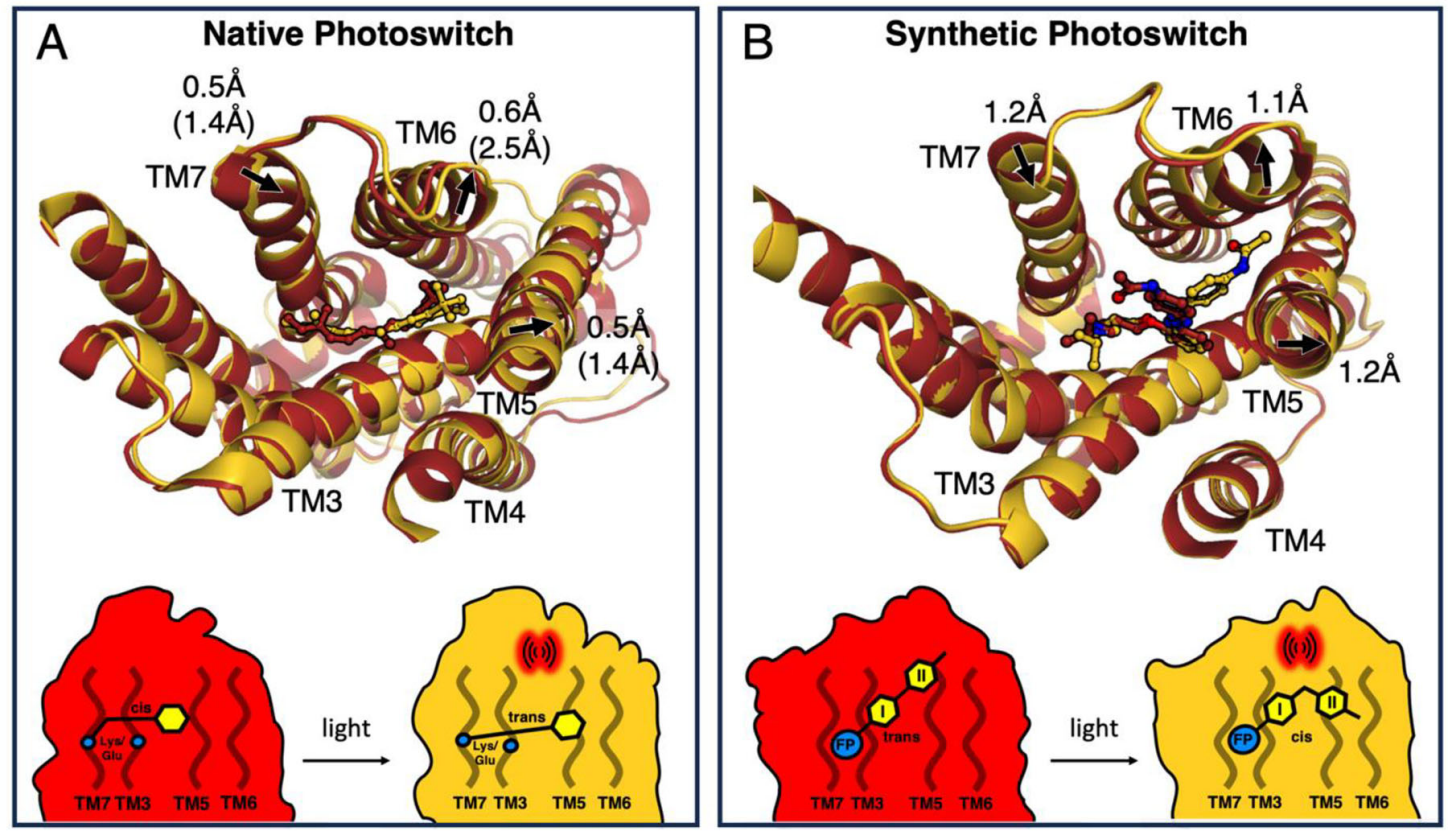
Comparison between native and synthetic photochemical efficacy switches. (A) The overlay of rhodopsin structures in the dark (pdb code: 7zbe) and lumi states (pdb code: 2hpy) shows how *cis*‐to‐*trans* isomerization relocates the β‐ionone ring of retinal. This leads to shifts in the positions of TM5, TM6, and TM7, which are the initial steps toward reaching the fully activated metarhodopsin‐II state (corresponding helix shifts in brackets). The schematic drawing shows the principal mechanisms of activity modulation with retinal covalently bound to Lys296^7.42^ in TM7 and interacting with TM3 by the Glu113^3.28^ counterion (blue circles). Light activation disrupts the local packing of the binding pocket through a shift of the β‐ionone ring (yellow hexagon) toward TM5 and TM6. (B) The overlay of the β_2_AR structures with photoazolol‐1 in the *trans* and *cis* configurations indicates similar shifts in TM5, TM6, and TM7. The schematic drawing highlights the similarities to rhodopsin with the fingerprint region (blue circle) bound between TM3 and TM7 and *trans*‐to‐*cis* isomerization shifting ring II of including the *p*‐acetamido group into a new position between TM5 and TM6, thereby interrupting the original packing of the binding pocket.

In order to understand the effect of photoazolol‐1 isomerization on activity, we compared the β_2_AR to visual rhodopsin, the GPCR activated through isomerization of its native ligand retinal within the binding pocket. Investigating the parallels could provide insights into the structural plasticity of GPCRs when reacting to a sudden change of a bound ligand and the mechanisms underlying the properties displayed by photoazolol‐1 (Figure 4). The overall structural changes after light excitation can be compared by overlaying the structures of dark [27], lumi [59], and activated meta‐II rhodopsin [60, 61] with those of our time‐resolved β_2_AR snapshots. Reminiscent of what we observed for photoazolol‐1, the light‐induced isomerization of the retinal polyene chain in rhodopsin places the β‐ionone ring between TM5 and TM6. Ultimately, this intercalation results in a shift of 2.6 Å in TM6 and a smaller 1.6 Å shift in TM5, while TM7 moves inwards by 1.2 Å, changes very comparable to our structure containing the fully isomerized photoazolol‐1 (Figures 4A and 4B). A further analogy in both receptors is the anchoring of the ligand to the protein at the part of the molecule most distant from TM5 and TM6. While this is achieved in the β_2_AR *via* the conserved interactions of Asp113^3.32^ in TM3 and Asn312^7.38^ in TM7 to the fingerprint region, the same is achieved in rhodopsin through electrostatic interactions to the retinal counterion residue Glu113^3.28^ in TM3 and the covalent Schiff base to Lys296^7.42^ in TM7. While these overall changes within the binding pockets are strikingly similar, there are also significant differences, including larger changes in TM3 in rhodopsin and the outward‐directed movement of the cytosolic ends of TM5 and TM6 that is characteristic for the G protein‐binding conformation. Further conformational changes may have been inhibited by crystal packing but most GPCRs, including the β_2_AR, need both an agonist and a signaling partner to adopt a fully active conformation [5, 9, 47].

Typically, GPCR agonists bind more tightly to the active state than the inactive state [62], and agonist binding is often considered to occur by conformational selection [10]. Unliganded opsin expresses the same behavior, where constitutively active variants can be directly activated by all‐*trans*‐retinal [63, 64]. However, native rhodopsin is exceptional among GPCRs as it functions through an induced fit mechanism, where the prebound *cis* retinal acts as an inverse agonist that is transformed by light to induce activation. This activation mechanism is surprisingly flexible, as the Lys residue covalently linking retinal can be moved to other locations while maintaining the ability to activate the G protein in a light‐dependent manner [65]. Covalently bound agonists have previously been used to study the mechanisms of β_2_AR activation [5, 66] and given the overall similarities in how Class A GPCRs are activated [35, 67], it seems feasible that disrupting the binding pocket and repositioning the ligand can modulate β_2_AR activation.

## 3 Conclusion

GPCR function is now recognized to involve complex conformational dynamics that extend far beyond classical two‐state activation models. Within this framework protein–ligand interaction dynamics play a central role in modulating receptor activity and the pharmacological potency of small‐molecule drugs. A key challenge in developing β‐blockers has been the design of ligands that selectively antagonize adrenergic receptors while minimizing off‐target or intrinsic pharmacological effects. Photopharmacology introduces light as a noninvasive tool for precise spatiotemporal control over drug activity. This is typically accomplished through photochemical affinity switches that undergo light‐dependent changes in binding affinity, enabling reversible control of receptor function via dynamic ligand binding and release.

Our structural findings suggest that the photoswitchable β‐blocker photoazolol‐1 modulates the β_2_AR through a distinct mechanism. Rather than dissociating upon photoisomerization, the ligand remains bound and induces local rearrangements within the orthosteric binding pocket. This conformational perturbation favors receptor activation, representing a conceptual shift from reversible occupancy‐based modulation (i.e. an affinity photoswitch) to internal structural repositioning of protein‐ligand interactions (i.e. an efficacy photoswitch). Such an approach to modulate receptor activity by light offers several advantages, including avoiding intramolecular competition between *cis* and *trans* isomers and reduced ligand loss due to diffusion or dilution, ultimately resulting in a more sustained and efficient light‐induced response.

These findings underscore the potential of photopharmacological ligands as mechanistic probes for understanding GPCR activation. When combined with time‐resolved structural approaches, this strategy provides high‐resolution mapping of the conformational landscapes that underlie ligand‐induced modulation of receptor activity. Iterative structural studies using chemically diverse, light‐responsive ligands may further reveal the intrinsic plasticity of GPCR binding pockets, informing the design of next‐generation therapeutics.

## Author Contributions

Conceptualization: MH, AI, XR and JS. Methodology: RS, QB, MT, RSI, XC, GG, PS, ADC, MTI, CS, TW, SM, and JB. Investigation: RS, QB, MT, ADC, MTI, HG, FS, HPS, MW, JC, MC, SH, MM, TM, YK, MA, TW, RC, CS, SM, JB, MK, XR, and JS. Visualization: RS, QB, MH, XR and JS. Supervision: JB, SM, MK, MH, XR, and JS. Writing – original draft: RS, XR and JS Writing – review and editing: all authors

## Conflicts of Interest

Some authors are employees of leadXpro AG, a company that offers services for GPCR drug design and develops its own lead compounds. The other authors declare no financial interests.

## Supporting information




**Supporting File 1**: anie71616‐sup‐0001‐SuppMat.pdf.

**Supporting File 2**: anie71616‐sup‐0002‐MovieS1.mov.

## Data Availability

Coordinates and structure factors have been deposited in the PDB database under accession codes 9RKF for the Dark state recorded at SwissFEL, 9RKG for the Light activated state recorded 10 s after activation at SwissFEL, 9RKH for the Dark state recorded at LCLS, and Finally, 9RKI for the Light activated state recorded 17 ns after activation at LCLS.

## References

[1] R. Santos , O. Ursu , A. Gaulton , et al., “A Comprehensive Map of Molecular Drug Targets,” Nature Reviews Drug Discovery 16 (2017): 19–34, 10.1038/nrd.2016.230. 27910877 PMC6314433

[2] W. I. Weis and B. K. Kobilka , “The Molecular Basis of G Protein–Coupled Receptor Activation,” Annual Review of Biochemistry 87 (2018): 897–919, 10.1146/annurev-biochem-060614-033910. PMC6535337 29925258

[3] V. Cherezov , D. M. Rosenbaum , M. A. Hanson , et al., “High‐Resolution Crystal Structure of an Engineered Human β_2_‐Adrenergic G Protein–Coupled Receptor,” Science 318 (2007): 1258–1265, 10.1126/science.1150577. 17962520 PMC2583103

[4] S. G. F. Rasmussen , H. J. Choi , D. M. Rosenbaum , et al., “Crystal Structure of the Human β_2_ Adrenergic G‐Protein‐Coupled Receptor,” Nature 450 (2007): 383–387, 10.1038/nature06325. 17952055

[5] D. M. Rosenbaum , C. Zhang , J. A. Lyons , et al., “Structure and Function of an Irreversible Agonist‐β_2_ Adrenoceptor Complex,” Nature 469 (2011): 236–240, 10.1038/nature09665. 21228876 PMC3074335

[6] L. Helfinger and C. G. Tate , Adrenoceptors, ed. J. G. Baker , M. C. Michel , and R. J. Summers , (Springer International Publishing, 2024), 13–26.

[7] M. M. Papasergi‐Scott , G. Pérez‐Hernández , H. Batebi , et al., “Time‐Resolved Cryo‐EM of G‐Protein Activation by a GPCR,” Nature 629 (2024): 1181–1191. 10.1038/s41586-024-07153-1 PMC11734571 38480881

[8] A. Manglik , T. H. Kim , M. Masureel , et al., “Structural Insights Into the Dynamic Process of β_2_‐Adrenergic Receptor Signaling,” Cell 162 (2015): 1431, 10.1016/j.cell.2015.08.045. PMC4441853 25981665

[9] R. O. Dror , D. H. Arlow , P. Maragakis , et al., “Activation Mechanism of the β_2_‐Adrenergic Receptor,” Proceedings of the National Academy of Sciences of the United States of America 108 (2011): 18684–18689, 10.1073/pnas.1110499108. 22031696 PMC3219117

[10] P. Conflitti , E. Lyman , M. S. P. Sansom , et al., “Functional Dynamics of G Protein‐Coupled Receptors Reveal New Routes for Drug Discovery,” Nature Reviews Drug Discovery 24 (2025): 251–275, 10.1038/s41573-024-01083-3. 39747671 PMC11968245

[11] K. Hüll , J. Morstein , and D. Trauner , “In Vivo Photopharmacology,” Chemical Reviews 118 (2018): 10710–10747, 10.1021/acs.chemrev.8b00037. 29985590

[12] P. Kobauri , F. J. Dekker , W. Szymanski , and B. L. Feringa , “Rational Design in Photopharmacology With Molecular Photoswitches,” Angewandte Chemie International Edition 62 (2023), 10.1002/anie.202300681. 37026576

[13] S. Panarello , X. Rovira , A. Llebaria , and X. Gómez‐Santacana , Molecular Photoswitches (Wiley‐VCH, 2022), 921–944, 10.1002/9783527827626.

[14] O. Bozovic , B. Jankovic , and P. Hamm , “Using Azobenzene Photocontrol to Set Proteins in Motion,” Nature Reviews Chemistry 6 (2022): 112–124, 10.1038/s41570-021-00338-6. 37117294

[15] M. J. Fuchter , “On the Promise of Photopharmacology Using Photoswitches: A Medicinal Chemist's Perspective,” Journal of Medicinal Chemistry 63 (2020): 11436–11447, 10.1021/acs.jmedchem.0c00629. 32511922

[16] A. Sink , H. Gerwe , H. Hübner , et al., ““Photo‐Adrenalines”: Photoswitchable β_2_‐Adrenergic Receptor Agonists as Molecular Probes for the Study of Spatiotemporal Adrenergic Signaling,” Chemistry (Weinheim An Der Bergstrasse, Germany) 30 (2024): e202303506, 10.1002/chem.202303506. 38212242

[17] R. Bosma , N. C. Dijon , Y. Zheng , et al., “Optical Control of the β_2_‐Adrenergic Receptor With Opto‐Prop‐2: A Cis‐Active Azobenzene Analog of Propranolol,” Iscience 25 (2022): 104882, 10.1016/j.isci.2022.104882. 36060054 PMC9436767

[18] A. Duran Corbera , J. Catena , M. Otero Vinas , A. Llebaria , and X. Rovira , “Photoswitchable Antagonists for a Precise Spatiotemporal Control of β_2_‐Adrenoceptors,” Journal of Medicinal Chemistry 63 (2020): 8458–8470.. 32686936 10.1021/acs.jmedchem.0c00831

[19] A. Duran‐Corbera , M. Faria , Y. Y. Ma , et al., “A Photoswitchable Ligand Targeting the β_1_‐Adrenoceptor Enables Light‐Control of the Cardiac Rhythm,” Angewandte Chemie International Edition 61 (2022), e202203449. 35608051 10.1002/anie.202203449 PMC9401038

[20] D. Prischich , A. M. J. Gomila , S. Milla‐Navarro , et al., “Adrenergic Modulation With Photochromic Ligands,” Angewandte Chemie (International ed in English) 60 (2021): 3625–3631, 10.1002/anie.202010553. 33103317

[21] M. P. Stapleton , “Sir James Black and Propranolol: The Role of the Basic Sciences in the History of Cardiovascular Pharmacology,” Texas Heart Institute Journal 24 (1997): 336–342. 9456487 PMC325477

[22] A. Aquila , M. S. Hunter , R. B. Doak , et al., “Time‐Resolved Protein Nanocrystallography Using an X‐Ray Free‐Electron Laser,” Optics Express 20 (2012): 2706, 10.1364/OE.20.002706. 22330507 PMC3413412

[23] J. Tenboer , S. Basu , N. Zatsepin , et al., “Time‐Resolved Serial Crystallography Captures High‐Resolution Intermediates of Photoactive Yellow Protein,” Science 346 (2014): 1242–1246, 10.1126/science.1259357. 25477465 PMC4361027

[24] T. Weinert , P. Skopintsev , D. James , et al., “Proton Uptake Mechanism in Bacteriorhodopsin Captured by Serial Synchrotron Crystallography,” Science 365 (2019): 61–65, 10.1126/science.aaw8634. 31273117

[25] G. Khusainov , J. Standfuss , and T. Weinert , “The Time Revolution in Macromolecular Crystallography,” Structure and Dynamics 11 (2024): 020901, 10.1063/4.0000247. PMC11015943 38616866

[26] G. Brändén and R. Neutze , “Advances and Challenges in Time‐Resolved Macromolecular Crystallography,” Science 373 (2021): 980, 10.1126/science.aba0954. 34446579

[27] T. Gruhl , T. Weinert , M. J. Rodrigues , et al., “Ultrafast Structural Changes Direct the First Molecular Events of Vision,” Nature 615 (2023): 939–944, 10.1038/s41586-023-05863-6. 36949205 PMC10060157

[28] B. Stauch and V. Cherezov , “Serial Femtosecond Crystallography of G Protein–Coupled Receptors,” Annual Review of Biophysics 47 (2018): 377–397, 10.1146/annurev-biophys-070317-033239. PMC6290114 29543504

[29] N. Vaidehi , R. Grisshammer , and C. G. Tate , “How Can Mutations Thermostabilize G‐Protein‐Coupled Receptors?,” Trends in Pharmacological Sciences 37 (2016): 37–46, 10.1016/j.tips.2015.09.005. 26547284 PMC4698185

[30] A. Ishchenko , B. Stauch , G. W. Han , et al., “Toward G Protein‐Coupled Receptor Structure‐Based Drug Design Using X‐Ray Lasers,” Iucrj 6 (2019): 1106–1119, 10.1107/S2052252519013137. 31709066 PMC6830214

[31] J. A. Ballesteros and H. Weinstein , Methods in Neurosciences, ed. S. C. Sealfon , (Academic Press, 1995), 366–428.

[32] T. Warne , R. Moukhametzianov , J. G. Baker , et al., “The Structural Basis for Agonist and Partial Agonist Action on a β_1_‐Adrenergic Receptor,” Nature 469 (2011): 241–244, 10.1038/nature09746. 21228877 PMC3023143

[33] L. Qu , Q. Zhou , Y. Xu , et al., “Structural Basis of the Diversity of Adrenergic Receptors,” Cell Reports 29 (2019): 2929–2935.e4, 10.1016/j.celrep.2019.10.088. 31801060

[34] C. D. Strader , I. S. Sigal , M. R. Candelore , E. Rands , W. S. Hill , and R. A. F. Dixon , “Conserved Aspartic Acid Residues 79 and 113 of the Beta‐Adrenergic Receptor Have Different Roles in Receptor Function,” Journal of Biological Chemistry 263 (1988): 10267–10271, 10.1016/S0021-9258(19)81509-0. 2899076

[35] F. M. Heydenreich , M. Marti‐Solano , M. Sandhu , B. K. Kobilka , M. Bouvier , and M. M. Babu , “Molecular Determinants of Ligand Efficacy and Potency in GPCR Signaling,” Science 382 (2023): 1378, 10.1126/science.adh1859. PMC7615523 38127743

[36] P. Nogly , T. Weinert , D. James , et al., “Retinal Isomerization in Bacteriorhodopsin Captured by a Femtosecond X‐Ray Laser,” Science 361 (2018), 10.1126/science.aat0094. 29903883

[37] P. Skopintsev , D. Ehrenberg , T. Weinert , et al., “Femtosecond‐to‐Millisecond Structural Changes in a Light‐Driven Sodium Pump,” Nature 583 (2020): 314–318, 10.1038/s41586-020-2307-8. 32499654

[38] T. Nägele , R. Hoche , W. Zinth , and J. Wachtveitl , “Femtosecond Photoisomerization of Cis‐Azobenzene,” Chemical Physics Letters 272 (1997): 489–495, 10.1016/S0009-2614(97)00531-9.

[39] I. K. Lednev , T. Q. Ye , R. E. Hester , and J. N. Moore , “Femtosecond Time‐Resolved UV−Visible Absorption Spectroscopy of trans ‐Azobenzene in Solution,” Journal of Physical Chemistry‐Us 100 (1996): 13338–13341, 10.1021/jp9610067.

[40] M. Wranik , T. Weinert , C. Slavov , et al., “Watching the Release of a Photopharmacological Drug From Tubulin Using Time‐Resolved Serial Crystallography,” Nature Communications 14 (2023): 903, 10.1038/s41467-023-36481-5. PMC9936131 36807348

[41] T. Weinert , M. Wranik , J. Church , et al., “Direct Observation of Coherent Azobenzene Photochemistry,” Research Square (2023), 10.21203/rs.3.rs-3490897/v1.

[42] H. Glover , T. Saßmannshausen , Q. Bertrand , et al., “Photoswitch Dissociation From a G Protein‐Coupled Receptor Resolved by Time‐Resolved Serial Crystallography,” Nature Communications 15 (2024), 10.1038/s41467-024-55109-w. PMC11686364 39738009

[43] Y. Kondo , C. Hatton , R. Cheng , et al., “Apo‐State Structure of the Metabotropic Glutamate Receptor 5 Transmembrane Domain Obtained Using a Photoswitchable Ligand,” Protein Science 34 (2025): e70104, 10.1002/pro.70104. 40521617 PMC12168131

[44] H. C. S. Chan , S. Filipek , and S. Yuan , “The Principles of Ligand Specificity on Beta‐2‐Adrenergic Receptor,” Scientific Reports 6 (2016), 34736, 10.1038/srep34736. 27703221 PMC5050457

[45] T. Warne , P. C. Edwards , A. G. W. Leslie , and C. G. Tate , “Crystal Structures of a Stabilized β_1_‐Adrenoceptor Bound to the Biased Agonists Bucindolol and Carvedilol,” Structure (London, England) 20 (2012): 841–849, 10.1016/j.str.2012.03.014. PMC3384003 22579251

[46] M. Masureel , Y. Z. Zou , L. P. Picard , et al., “Structural Insights Into Binding Specificity, Efficacy and Bias of a β_2_AR Partial Agonist,” Nature Chemical Biology 14 (2018): 1059–1066, 10.1038/s41589-018-0145-x. 30327561 PMC6197491

[47] S. G. F. Rasmussen , B. T. DeVree , Y. Z. Zou , et al., “Crystal Structure of the β_2_ Adrenergic Receptor–Gs Protein Complex,” Nature 477 (2011): 549–555, 10.1038/nature10361. 21772288 PMC3184188

[48] G. Y. Mitronova , G. Lukinavicius , A. N. Butkevich , et al., “High‐Affinity Functional Fluorescent Ligands for Human β‐Adrenoceptors,” Scientific Reports‐Uk 7 (2017): 12319. 10.1038/s41598-017-12468-3 PMC5614969 28951558

[49] J. Klarenbeek , J. Goedhart , A. van Batenburg , D. Groenewald , and K. Jalink , “Fourth‐Generation Epac‐Based FRET Sensors for cAMP Feature Exceptional Brightness, Photostability and Dynamic Range: Characterization of Dedicated Sensors for FLIM, for Ratiometry and With High Affinity,” PLoS ONE 10 (2015): e0122513. 25875503 10.1371/journal.pone.0122513 PMC4397040

[50] X. Xu , J. Shonberg , J. Kaindl , et al., “Constrained Catecholamines Gain β_2_AR Selectivity Through Allosteric Effects on Pocket Dynamics,” Nature Communications 14 (2023): 2138, 10.1038/s41467-023-37808-y. PMC10104803 37059717

[51] X. Xu , J. Kaindl , M. J. Clark , et al., “Binding Pathway Determines Norepinephrine Selectivity for the Human β_1_AR Over β_2_AR,” Cell Research 31 (2021): 569–579, 10.1038/s41422-020-00424-2. 33093660 PMC8089101

[52] Y. Cao , S. Shi , S. A. H. Does , et al., “Photo‐Clenbuterol: Optical Control of β_2_‐Adrenergic Receptor Signaling by Photoswitchable Ligand Efficacy,” Journal of Medicinal Chemistry 68 (2025): 12911–12924, 10.1021/acs.jmedchem.5c00792. 40492834 PMC12225454

[53] U. Wirth , E. Neu , D. Provasi , et al., “Synthesis and Characterization of Photoswitchable Covalent Ligands for the β_2_‐Adrenoceptor,” Angewandte Chemie International Edition 64 (2025), 10.1002/anie.202424038. PMC12144893 40192602

[54] Y. Han , P. Wang , Z. Liao , et al., “Exploring the Divergence of Rare Earth Trade Networks With a Global Simulation Model,” Iscience 28 (2025): 114122, 10.1016/j.isci.2025.114122. 41146717 PMC12554209

[55] B. I. Gaiser , M. Danielsen , X. Xu , et al., “Bitopic Ligands Support the Presence of a Metastable Binding Site at the β 2 Adrenergic Receptor,” Journal of Medicinal Chemistry 67 (2024): 11053–11068, 10.1021/acs.jmedchem.4c00578. 38952152

[56] A. C. Kruse , B. K. Kobilka , D. Gautam , P. M. Sexton , A. Christopoulos , and J. Wess , “Muscarinic Acetylcholine Receptors: Novel Opportunities for Drug Development,” Nature Reviews Drug Discovery 13 (2014): 549–560, 10.1038/nrd4295. 24903776 PMC5818261

[57] D. A. Sykes , C. Parry , J. Reilly , P. Wright , R. A. Fairhurst , and S. J. Charlton , “Observed Drug‐Receptor Association Rates Are Governed by Membrane Affinity: The Importance of Establishing “Micro‐Pharmacokinetic/Pharmacodynamic Relationships” at the β_2_‐Adrenoceptor,” Molecular Pharmacology 85 (2014): 608–617, 10.1124/mol.113.090209. 24476583

[58] C. J. Dickson , V. Hornak , C. Velez‐Vega , et al., “Uncoupling the Structure–Activity Relationships of β_2_ Adrenergic Receptor Ligands From Membrane Binding,” Journal of Medicinal Chemistry 59 (2016): 5780–5789, 10.1021/acs.jmedchem.6b00358. 27239696

[59] H. Nakamichi and T. Okada , “Local Peptide Movement in the Photoreaction Intermediate of Rhodopsin,” PNAS 103 (2006): 12729–12734, 10.1073/pnas.0601765103. 16908857 PMC1562544

[60] H.‐W. Choe , Y. J. Kim , J. H. Park , et al., “Crystal Structure of Metarhodopsin II,” Nature 471 (2011): 651–655, 10.1038/nature09789. 21389988

[61] X. Deupi , P. Edwards , A. Singhal , et al., “Stabilized G Protein Binding Site in the Structure of Constitutively Active Metarhodopsin‐II,” PNAS 109 (2012): 119–124, 10.1073/pnas.1114089108. 22198838 PMC3252945

[62] R. S. Kent , A. De Lean , and R. J. Lefkowitz , “A Quantitative Analysis of Beta‐Adrenergic Receptor Interactions: Resolution of High and Low Affinity States of the Receptor by Computer Modeling of Ligand Binding Data,” Molecular Pharmacology 17 (1980): 14–23, 10.1016/S0026-895X(25)14062-5. 6104284

[63] M. Han , S. O. Smith , and T. P. Sakmar , “Constitutive Activation of Opsin by Mutation of Methionine 257 on Transmembrane Helix 6,” Biochemistry 37 (1998): 8253–8261, 10.1021/bi980147r. 9609722

[64] M. Han , S. W. Lin , M. Minkova , S. O. Smith , and T. P. Sakmar , “Functional Interaction of Transmembrane Helices 3 and 6 in Rhodopsin,” Journal of Biological Chemistry 271 (1996): 32337–32342, 10.1074/jbc.271.50.32337. 8943296

[65] E. L. Devine , D. D. Oprian , and D. L. Theobald , “Relocating the Active‐Site Lysine in Rhodopsin and Implications for Evolution of Retinylidene Proteins,” Proceedings of the National Academy of Sciences of the United States of America 110 (2013): 13351–13355, 10.1073/pnas.1306826110. 23904486 PMC3746867

[66] D. Weichert , A. C. Kruse , A. Manglik , et al., “Covalent Agonists for Studying G Protein‐Coupled Receptor Activation,” Proceedings of the National Academy of Sciences of the United States of America 111 (2014): 10744–10748, 10.1073/pnas.1410415111. 25006259 PMC4115510

[67] Q. T. Zhou , D. H. Yang , M. Wu , et al., “Common Activation Mechanism of Class A GPCRs,” Elife 8 (2019): e50279. 31855179 10.7554/eLife.50279 PMC6954041

