# Supplementary material for: Structural Mechanism of an Efficacy Photoswitch Targeting the β2‐adrenergic Receptor
Source: Angew Chem Int Ed Engl. 2026 Mar 18;65(18):e17995. doi: 10.1002/anie.202517995 (PMC13110768; doi:10.1002/anie.202517995)
Supplement: Supplementary file 1 — Supporting File 1: anie71616‐sup‐0001‐SuppMat.pdf. [file ANIE-65-e17995-s002.pdf]

Supporting Information

Structural Mechanism of an Efficacy Photoswitch Targeting the β_2-adrenergic receptor

Robin Stipp[a],#, Quentin Bertrand[a],#, Matilde Trabuco[b], Anna Duran-Corbera[c], Maria Tindara Ignazzitto[c], Hannah Glover[a],[b], Fabienne Stierli[a], Juanlo Catena[c],[d], Melissa Carrillo[a], Sina Hartmann[a], Hans-Peter Seidel[a], Matthias Mulder[a], Thomas Mason[a], Yasushi Kondo[a], Maximillian Wranik[a],[e], Martin Appleby[f], Christoph Sager[b], Raymond Sierra[g], Gregory Gate[g], Pamela Schleissner[g], Xinxin Cheng[g], Tobias Weinert[a], Robert Cheng[b], Sandra Mous[g], John H. Beale[f], Michal Kepa[a], Amadeu Llebaria[c], Michael Hennig[b], Xavier Rovira*,[c], Joerg Standfuss*,[a],[h]

Table of Contents

Supporting Figures and Tables

Supporting Movie caption

Material and Methods

References

WILEY-VCH

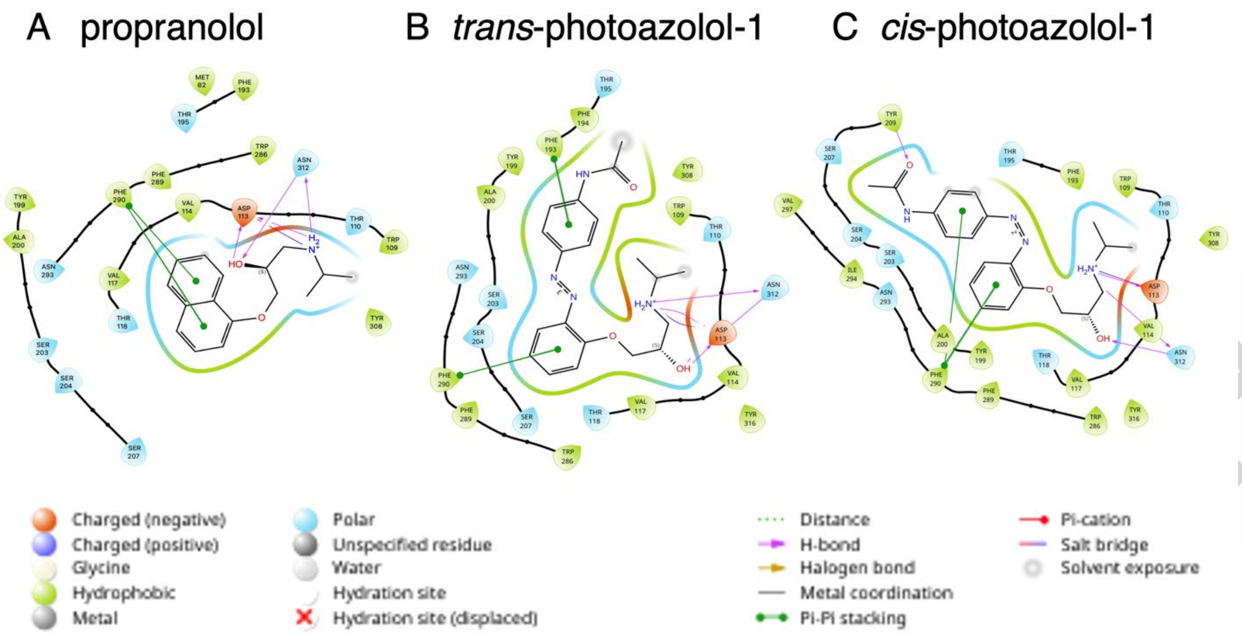

D Ligand Binding Energy

	trans-photoazolol-1	*cis*-photoazolol-1
Coulomb	-62.02	-63.16
Covalent binding	-2.43	2.36
Van der Waals	-47.05	-48.12
Lipophilic	-28.94	-26.21
G.B.E.S.	69.05	67.41
H-bond correction	-3.25	-3.73
π-stacking correction	-2.63	-2.22
Total energy	-77.27	-73.68

Figure S1: Ligand binding energy and interaction patterns. 2D projection of the receptor-ligand interactions for propranolol (**A**), photoazolol-1 in its (**B**) dark-equilibrated *trans* and (**C**) relaxed *cis* configurations 10 s after activation. The type of interaction and the participating protein residues are color-coded according to the shown legend. Lines indicate important interactions. Presentations were generated using SiteMap in Maestro[50]. (**D**) Ligand binding energies calculated using MM/GPSA (Molecular Mechanics/Generalized Born Surface Area) in Maestro [50]. The relative energies of photoazolol-1 in the dark (left) and 10 s structures (right) compared to the relaxed ligand in solvent are shown. In both cases, ligand-receptor interactions remain highly favorable, with the cis-isoform exhibiting a slightly lower total energy difference. Loss of polar interactions with Asn293$^{6.55}$ and Ser204$^{5.43}$ is compensated by stronger interactions with TM5, notably a hydrogen bond with Tyr209$^{5.48}$. Ligand intercalation between helices 5 and 6 enhances van der Waals interactions, bringing ring II into close contact with both helices. The hydrophobic acetate group also shifts into a hydrophobic receptor region, reducing unfavorable G.B.E.S. (Generalized Born electrostatic solvation) energy and increasing the contact area. A loss of lipophilic interactions partially offsets these favorable changes post-*trans*-*cis* isomerization, due to helix reorganization, increasing polarity near the lipophilic isopropyl residue in the molecular fingerprint. In both cases, the ligand-receptor complex remains in a significantly lower energy state than the unbound ligand and receptor, aligning with the observed ligand retention in the binding pocket several seconds after isomerization.

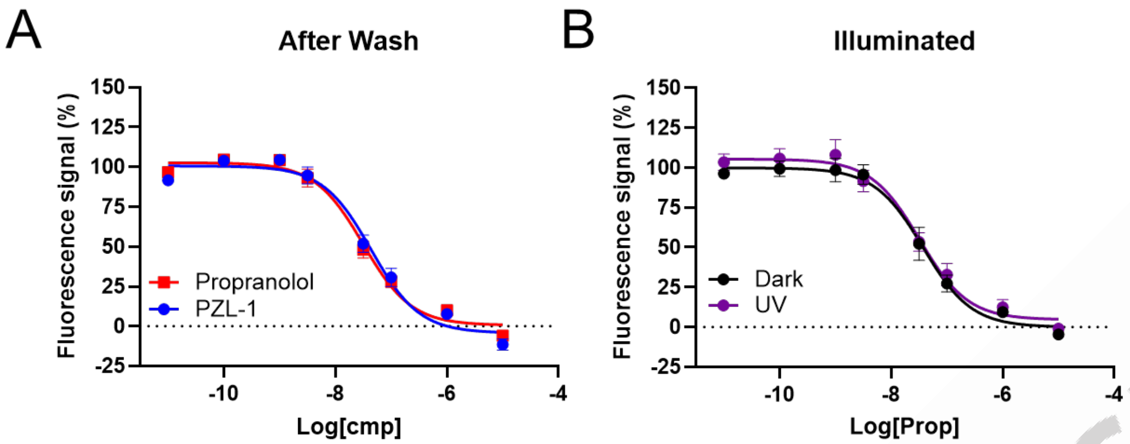

Figure S2: Ligand binding to the β$_2$AR. (**A**) Competitive binding curves of photoazolol-1 and propranolol with a constant concentration of the fluorescent ligand carazolol-KK114 (100 nM). Measurements were conducted right after the samples had been thoroughly washed. (**B**) Competitive binding curves of propranolol with a constant concentration of the fluorescent ligand carazolol-KK114 (100 nM). Measurements were performed 1 hour after the thorough washing. Data are shown as the mean ± standard error of the mean (SEM) of three independent experiments in duplicate.

WILEY-VCH

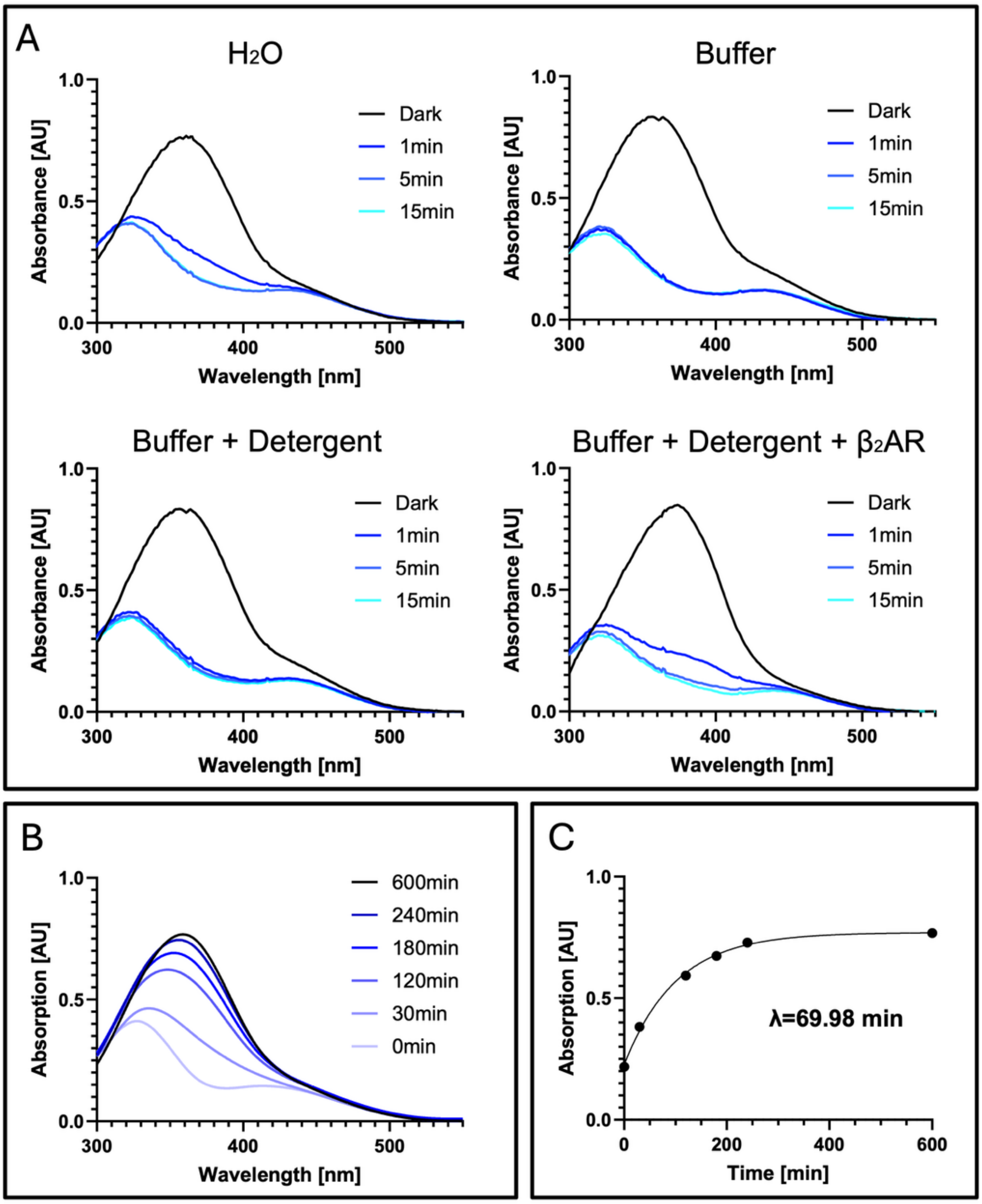

Figure S3: Spectroscopic analysis of photoazolol-1 isomerization and relaxation. (**A**) Photoazolol-1 was dissolved in different conditions (pure water, protein storage buffer without detergent, with detergent and with detergent and an 2-fold excess of β_2AR. All samples were illuminated with a 365 nm photodiode for 1, 5 and 15 minutes or were left unilluminated. (**B**) To investigate photoazolol-1 relaxation the ligand was illuminated for 15 minutes using a 365 nm photodiode to ensure maximal conversion to the *cis*-isomer and subsequently stored under exclusion of light at 20°C. Spectra were recorded after 0 min, 30 min, 120 min, 180 min, 240 min and 600 min. Lines were smoothened for increased clarity using the “fit spline” function of GraphPad Prism. (**C**) Thermal relaxation was modeled as a one-phase decay using the change of absorbance at 360 nm.

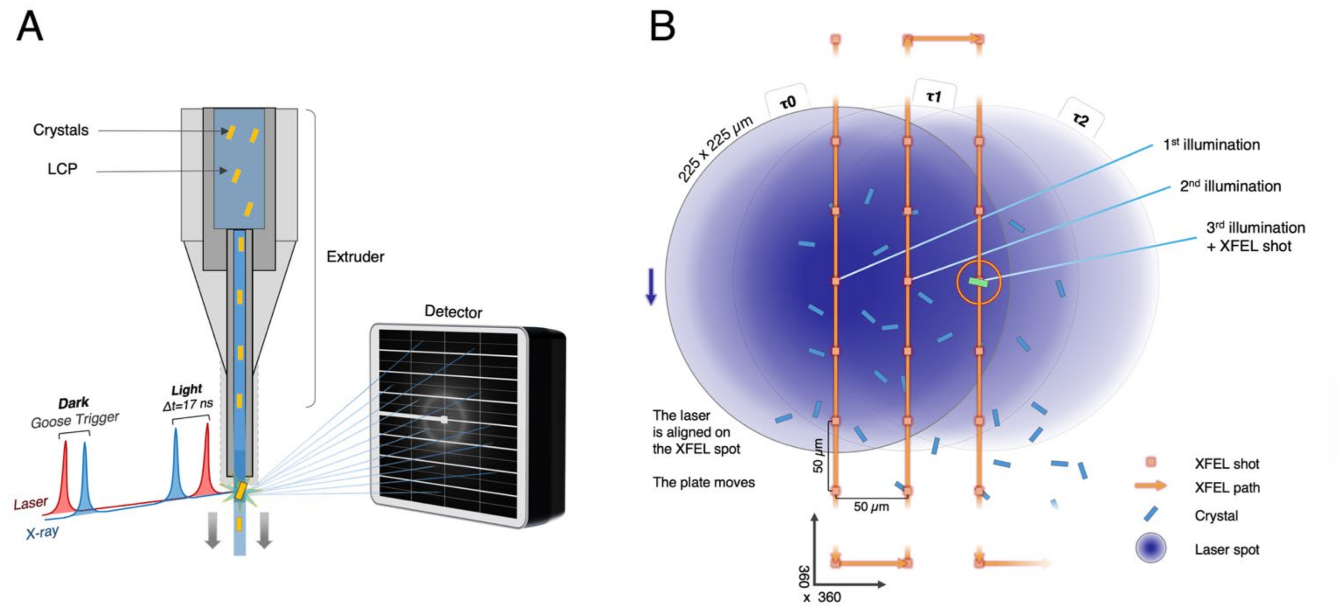

Figure S4: Experimental setups used for time-resolved data collection. (**A**) The 17 ns data was collected at the Coherent X-ray Imaging (CXI) beamline at the LCLS. The classical TR-SFX setup relies on a high-viscosity extruder (HVE), similar to what we had used there previously[26a, 51], but relying on a nanosecond laser. The light data were recorded by triggering a nanosecond laser 17 ns prior the X-ray pulse, while the dark data were collected with a "duck and goose" approach where the laser was triggered after the X-ray pulse. (**B**) The data from SwissFEL were collected at the new Cristallina fixed-target station using sheet-on-sheet devices[52]. Each chip was probed by the XFEL pulses in a 360 x 360 matrix (columns x rows), with each column taking 3.6 s to complete at the 100 Hz repetition rate of SwissFEL. During data collection, the sample was illuminated by a laser diode with a spot size of ≅ 225 μm diameter. Considering 50 μm distance between XFEL shots, at a time **τ**0, the laser shines up to the column c_{+2} where is our crystal of interest (green crystal in the figure). Based on this experimental setup, the time between the first illumination and the X-ray probe is thus approximately 10 s. The main advantages of the solid-support system are a sample efficient data collection and the ability to probe longer time delays than are possible with extruder-based setups. The drawbacks are lower time accuracy and higher chance to measure a mixture of intermediates compared to the accurate pump-probe setup at LCLS.

WILEY

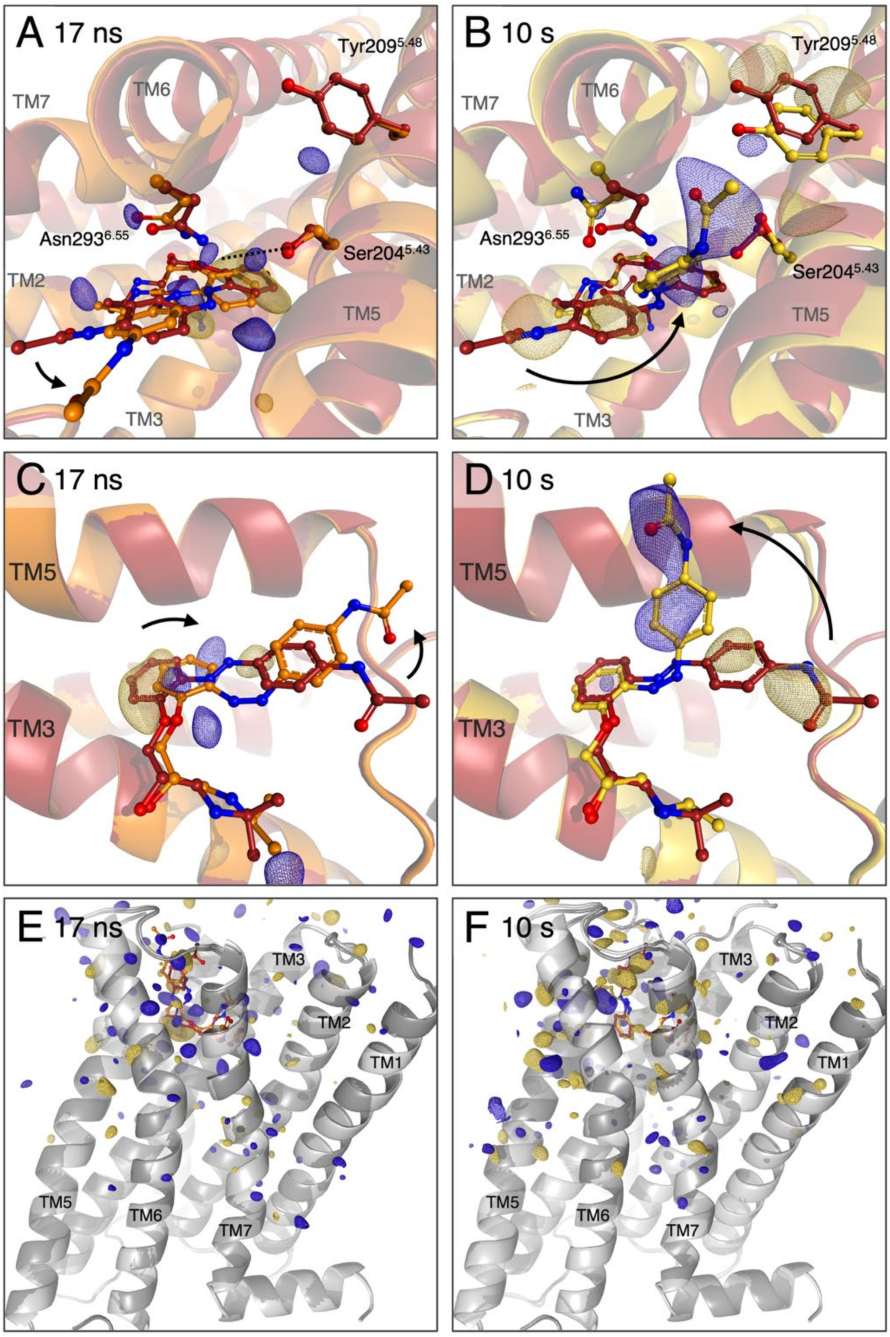

Figure S5: Difference electron density maps. (**A** and **C**) Early rearrangements in response to photoazolol-1 isomerization. The F_o(17ns) − F_o(dark) electron density map indicates isomerization and a shift in the position of photoazolol-1 shortly after photoactivation. This transient *cis* configuration induces only a little movement in the binding pocket. (**B** and **D**) Adaptation of the binding pocket upon photoazolol-1 relaxation. The F_o(~10s) − F_o(dark) electron density map indicates relaxation of the initially strained *cis* configuration, including a repositioning of the *p*-acetamido substituted ring **II** of photoazolol-1 (arrows). The new position in between TM5 and TM6 interrupts the interaction between Asn293$^{6.55}$ and Ser204$^{5.43}$. (**E** and **F**) F_o(light) − F_o(dark) electron density maps carved at 3 angstroms around the entirety of β_{2A}R models for respectively 17 ns and 10 s. The majority of the signal is visible around the ligand binding pocket, and the stronger peaks are located on the ligand. All maps are represented at 3 sigma, with positive density in blue and negative density in yellow.

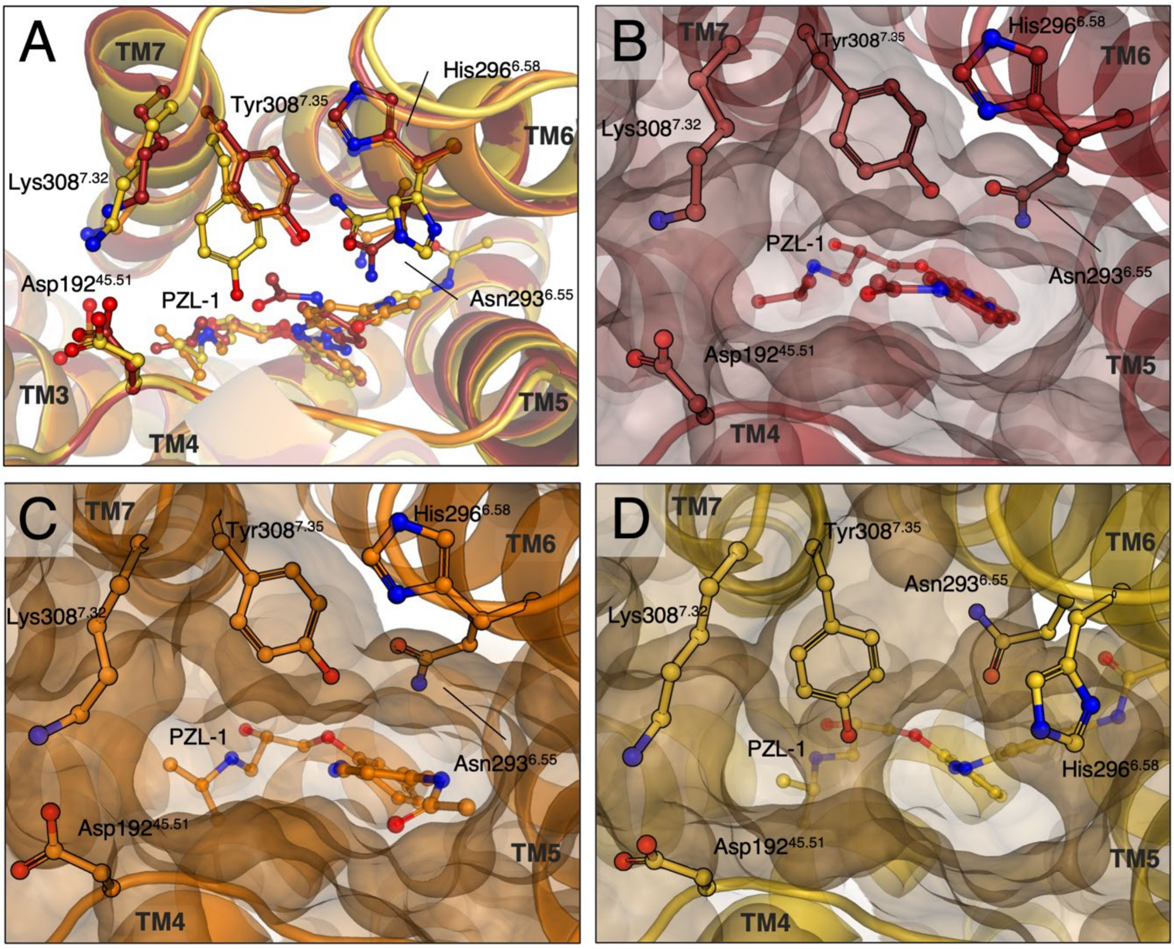

Figure S6: Reorganization of residues above the binding pocket upon photoazolol-1 isomerization. (A) Superposition of photoazolol-1–bound structures (cartoon representation) of the dark-adapted receptor (red), and the receptor 17 ns (orange) and ~10 s (gold) after photoisomerization. The reorganization of photoazolol-1 (PZL-1) and interacting residues (ball-and-stick representation) is shown. Carbon atoms are colored according to the respective structure; oxygen atoms are shown in red and nitrogen atoms in blue. (**B**) Shape of the ligand binding pocket in dark-adapted receptor containing *trans*-photoazolol-1, (**C**) with the strained *cis*-photoazolol-1 conformation resolved 17 ns after photoactivation and (**D**) the repositioned *cis*-photoazolol-1 in the second range.

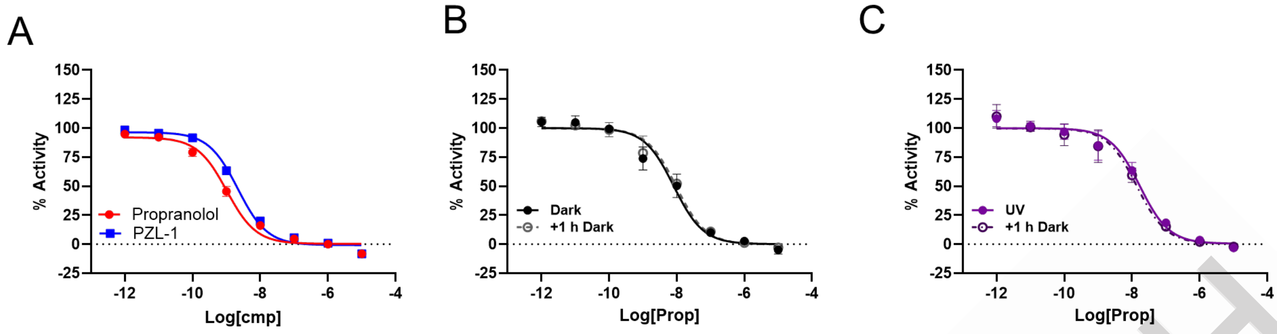

Figure S7: Ligand activity on β_2AR before washing and after washing in the presence of a low concentration of agonist. All experiments were performed with a constant concentration of the agonist cimaterol (10 nM). (**A**) Dose-response curves of PZL-1 and propranolol before the samples had been thoroughly washed. (**B**) Dose-response curves of propranolol 15 min after washing kept in the dark (solid black lines), and after an additional 1-hour incubation in the dark (dashed black lines). (**C**) Dose-response curves of propranolol 15 min after washing kept under light at 380 nm (solid violet lines) and after an additional 1-hour incubation in the dark (dashed violet lines). Data are shown as the mean ± SEM of three independent experiments in duplicate.

WILEY-VCH

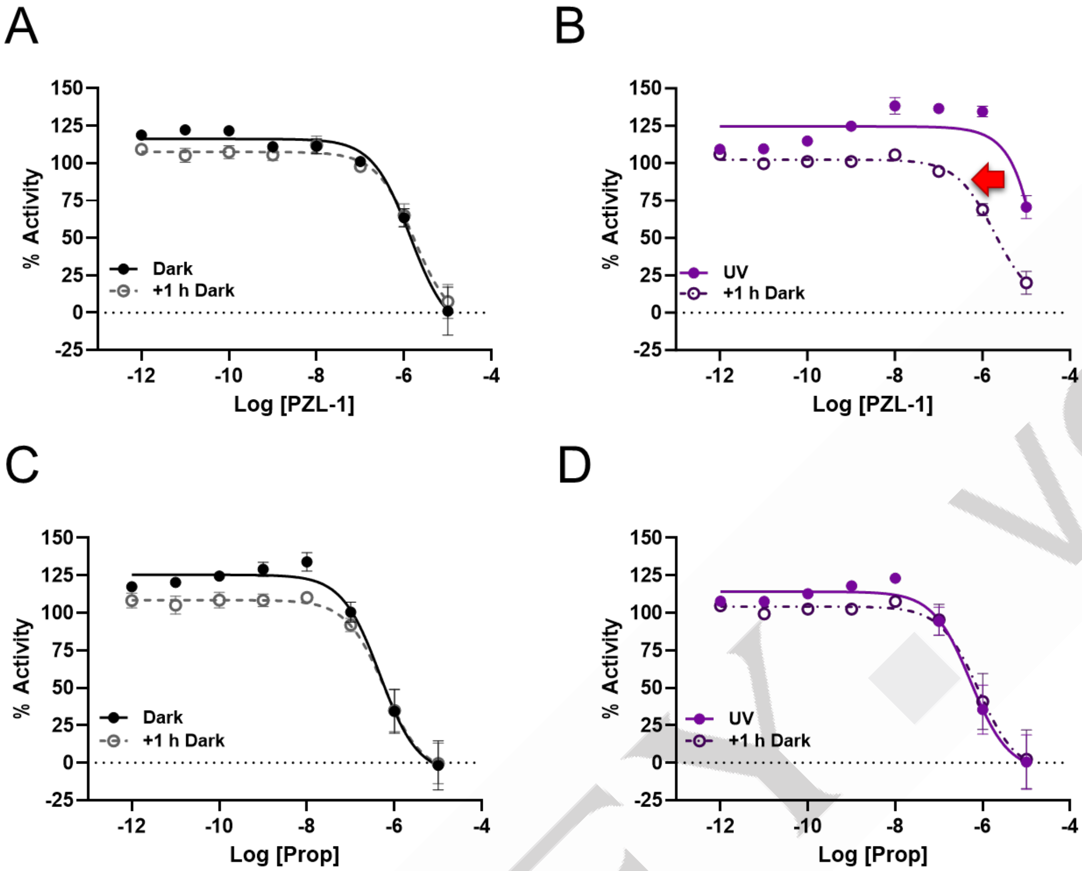

Figure S8: Ligands function on β$_2$AR in the presence of high concentrations of agonist. All experiments were performed with a constant concentration of the agonist cimaterol (1 µM). (**A**) Dose-response curves of photoazolol-1 15 min after washing kept in the dark (solid black lines), and after an additional 1-hour incubation in the dark (dashed black lines). (**B**) Dose-response curves of photoazolol 15 min after washing kept under light at 380 nm (solid violet lines) and after an additional 1-hour incubation in the dark (dashed violet lines). (**C**) Dose-response curves of propranolol 15 min after washing kept in the dark (solid black lines), and after an additional 1-hour incubation in the dark (dashed black lines). (**D**) Dose-response curves of propranolol 15 min after washing kept under light at 380 nm (solid violet lines) and after an additional 1-hour incubation in the dark (dashed violet lines). Data are shown as the mean ± SEM of three independent experiments in duplicate.

WILEY-VCH

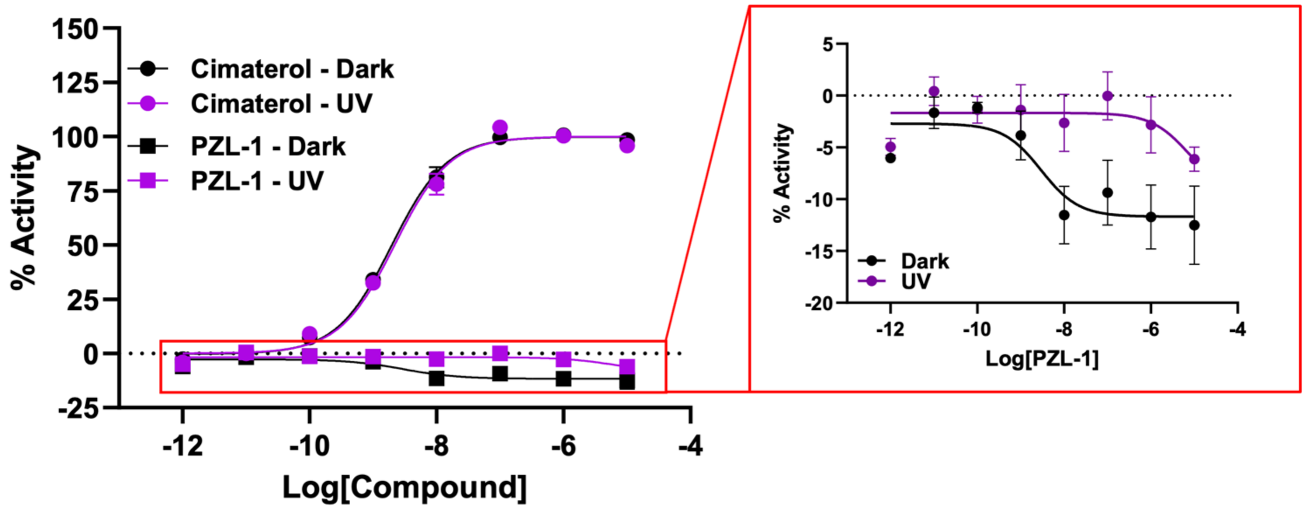

Figure S9: Photoazolol-1 and cimaterol activity in cells overexpressing β_2AR. Dose–response curves for cimaterol and photoazolol-1 were performed using EPAC cAMP FRET reporter assays in HEK 293 cells transfected with β_2AR. Data for the dose-response curves of photoazolol-1 were collected 15 min after washing, either in the dark or under illumination at 380 nm. Cimaterol was added before and after the washing steps and kept in the dark or under illumination at 380 nm. Data are shown as the mean ± standard error of the mean (SEM) from three independent experiments performed in duplicate. Data were normalized to the FRET ratio obtained with buffer (0%) and to the maximal response obtained with cimaterol (100%).

WILEY

Movie S1: Morph between β_2AR structures containing dark-adapted and light-activated photoazolol-1. This morph between the dark (red), 17 ns (orange), and 10 s (gold) structures shows how the β_2AR binding pocket adapts during the *trans*-to-*cis* isomerization of photoazolol-1, viewed from the extracellular side (left) and the membrane plane (right). For comparison, the dark-state structure is shown in transparent grey throughout the movie.

WILEY-VCH

Dataset	*SwissFEL – Cristallina – 10s*		*LCLS – CXI – 17ns*	
	Dark	Light	Dark	Light
**Data Collection**				
Space group	*C121*			
Unit cell *a, b, c* (Å) / α , β , γ (°)	111.94, 172.23, 41.23 90.0, 106.2, 90.0			
Indexed crystals	88 849	84 524	55 816	40 650
**Overall Statistics (High-Resolution Statistics)**				
Resolution (Å)	53.75 – 2.45 (2.54 – 2.45)	53.75 – 2.45 (2.54 – 2.45)	28.33 – 2.5 (2.59 – 2.5)	28.33 – 2.6 (2.69 – 2.6)
Total Reflections	13 464 666	12 834 526	3 979 447	2 804 141
Unique Reflections	27 481 (2741)	27 481 (2741)	25 868 (2609)	22 987 (2304)
<*I/s(I)*>	8.40 (0.75)	8.27 (0.74)	6.43 (0.54)	6.16 (0.63)
CC^*	0.998 (0.626)	0.998 (0.669)	0.996 (0.767)	0.995 (0.711)
R_{split} (%)	8.16 (143.62)	8.35 (142.77)	12.35 (153.68)	13.80 (133.78)
Completeness (%)	100 (100)	100 (100)	100 (100)	100 (100)
Multiplicity	496 (125)	467 (125)	153 (104)	121 (80)
**Refinement**				
Resolution Range	45.60 – 2.45	45.60 – 2.45	28.33 – 2.5	28.33 – 2.60
No. Reflections	23 701	23 487	25 813	22 971
R_{work} / R_{free} (%)	18.09 / 21.86	18.31 / 21.69	19.31 / 21.83	19.59 / 23.40
**No. Atoms**				
Protein	3556	7107	3560	7094
Ligand	342	696	323	667
Solvent	31	4	26	33
**B Factors**				
Protein	76.74	78.34	79.54	93.17
Ligand	95.68	100.63	98.41	109.33
Solvent	67.35	52.42	72.19	37.97
**R.m.s Deviations**				
Bond Lengths (Å)	0.009	0.004	0.005	0.004
Bond Angles (°)	0.690	0.782	0.898	0.853
**Ramachandran**				
Favoured (%)	97.28	97.28	97.73	97.73
Allowed (%)	2.72	2.72	2.27	2.29
Outliers (%)	0.00	0.00	0.00	0.00
PDB	**9RKF**	**9RKG**	**9RKH**	**9RKI**

Table S1: Crystallographic data and refinement statistics.

WILEY-VCH

Materials and Methods

Construct and β$_2$AR expression

A previously described construct was used to obtain a version of the β$_2$-adrenergic receptor suitable for crystallization and ensure suitable protein expression and stability[22, 53]. Compared to the human wtβ$_2$AR, the following modifications were performed on the construct (UniProt ID P07550): The C-terminus was truncated at residue 384 and ICL3 (residues 231-262) was replaced with a cysteine-free T4-lysozyme, improving stability and crystallizability. Additionally, a point mutation (E122W)[54] was inserted to improve stability and protein yield. The construct was expressed in *Trichoplusia ni* Hi5 cells using the FlashBAC system. The cells were grown in Sf900 II media at a density of 2*10^6 cells/ml at 27°C under constant shaking. Following Bacmid infection using 1% VOI, the cells were incubated for 72h before harvesting at 3500xg for 10min and flash freezing in liquid nitrogen.

Protein purification

Membrane preparation consisted of resuspension of the thawed cell pellet in hypotonic buffer (10 mM HEPES pH 7.5, 20 mM KCL, 10 mM $MgCl_2$, supplemented with Pierce^{TM} Protease inhibitor tablets) in a 1:6 ratio of pellet mass to buffer volume. The cell membrane was cracked using a Dounce homogenizer (20 strokes) followed by ultracentrifugation at 200,000x g for 30 min. The supernatant was discarded, and the pellet was resuspended in fresh hypotonic buffer using an Ultra-Turrax T25 basic (10 seconds of mixing), followed by ultracentrifugation. This washing method was repeated twice using hypertonic buffer (10 mM HEPES pH 7.5, 20 mM KCl, 10 mM $MgCl_2$, 1 M NaCl, supplemented with protease inhibitor). After the last centrifugation step, the pellet was flash-frozen and stored at -80°C. The purification method is based on previous work [55] but was modified for increased efficiency and purity. The membrane pellet was thawed on ice and resuspended in solubilization buffer (25 mM HEPES pH 7.5, 300 mM NaCl, 5 mM imidazole pH 7.5). To the solution, 2 mg/mL iodoacetamide, protease inhibitor tablets and DNase I was added. Lauryl Maltose neopentyl glycol (LMNG) and cholesteryl hemisuccinate (CHS) were introduced to a final concentration of 1% and 0.2% (w/v), respectively. The solution was stirred at 300 rpm and 4°C for 90 min and subsequently clarified by ultracentrifugation (200,000 g, 40 min). The supernatant was combined with TALON-beads by Takara Bio (previously equilibrated to the solubilization buffer) and incubated at 100 rpm and 4°C, stirring for 1 hr. 1 mL of TALON beads were used for every 100 mL of solubilization supernatant. After incubation, the beads were packed into an XK16 column and washed with 10 column volumes (CV) of wash buffer I [50 mM HEPES pH 7.5, 300 mM NaCl, 20 mM imidazole pH 7.5, 0.03% (w/v) LMNG, 0.003% (w/v) CHS], followed by 5 CV of wash buffer II [50 mM HEPES pH 7.5, 300 mM NaCl, 50 mM imidazole pH 7.5, 0.03% (w/v) LMNG, 0.003% (w/v) CHS] at 2 mL/min each. The protein was eluted from the protein with elution buffer [25 mM HEPES pH 7.5, 300 mM NaCl, 300mM imidazole pH 7.5, 10% (v/v) glycerol, 0.003% (w/v) LMNG, 0.0003% (w/v) CHS] at a flowrate of 0.5. mL/min. The Buffer was exchanged to storage buffer [25 mM HEPES pH 7.5, 300 mM NaCl, 10% (v/v) glycerol, 0.003% (w/v) LMNG, 0.0003% (w/v) CHS] using PD-10 desalting columns and concentrated to 30 mg/mL using Amicon concentrators at 1200 g. The purified protein was flash-frozen in liquid nitrogen and stored at -80°C.

Crystallization

In preparation for crystallization, samples of β$_2$AR in storage buffer at a concentration of 30 mg/mL were thawed and supplemented with 2 mM of photoazolol-1 (PZL-1). Two parts of the protein solution were mixed with three parts of molten monoolein [supplemented with 10% (w/v) cholesterol] and homogenized until a clear phase was generated. Initial LCP screening was done using a Gryphon robot (ARI) in 96-well glass sandwich plates (Laminex). 40 nL of LCP were placed in each well and covered with 800 nL of mother liquor supplemented with 10 µm photoazolol-1. Several commercial screens were used to find initial crystallization conditions. After optimization, crystallization was scaled up using a modified in-well crystallization method[56] in EasyXtal plates (Molecular Dynamics). This allowed reproducible production of large amounts of crystal-laden LCP needed for serial crystallography. 10 µL of LCP were added to wells containing 300 µl of mother liquor [100 mM tri-sodium citrate pH 6.2, 245 mM Li_2SO_4, 32% (w/v) PEG 350 MME, 10 µM photoazolol-1] at a 1:30 LCP:mother liquor ratio and the plate was shielded against ambient illumination with aluminum foil and incubated at 16°C. First crystals appeared within 24 hr and were fully grown after 96 hr. The crystal-laden LCP was harvested and pooled in 500 µL Hamilton syringes and topped off with small amounts of mother liquor to avoid drying. All crystallization steps were conducted under red-light conditions and at 20°C ambient temperature.

WILEY-VCH

Time-resolved data collection

The 17 ns time-point was collected using the coherent X-ray imaging (CXI) beamline at the Linac Coherent Light Source (LCLS). Samples were delivered to the X-ray using high viscosity extrusion[57]. The crystal-laden LCP was modified by adding PEG2000 and monoolein to reach a viscosity suitable for jetting. The final jetting phase contained 62.5% crystal-laden LCP, 34.7% monoolein and 2.8% PEG2000. Jetting was conducted with a 75 µm glass capillary nozzle at 21.5 µm/ms, corresponding to 180 µm between consecutive XFEL pulses. Data was collected using a Jungfrau 4M detector at 120 Hz with an X-ray focused to about 2x2 µm (horizontal x vertical) FWHM at 8.8keV. A pump laser diode (EKSPLA NL-204) at 355 nm was used to trigger the isomerization reaction. The laser spot size was 88 µm ($1/e^2$) and delivered a fluence of 216 mJ/cm^2 in 8 ns pulses. The light dataset was recorded with a 1:1 dark to light ratio with the dark images being goose triggered, meaning that the laser arrives on the interaction zone 40 ns after the X-ray pulse. True-dark data were recorded before the pump-probe experiment for control. The slow time-point (~10 s) was collected at the Cristallina experimental station of SwissFEL. Here, samples were delivered with solid support, using Sheet-On-Sheet chips[58]. Each chip was loaded with a volume of 25 µl of LCP sample and probed with 360x360 (columns x rows) XFEL pulses. Data were collected using a Jungfrau 8M detector at 100 Hz with an X-ray focused to 5x5 µm FWHM at 12.044 keV. A laser diode at 405 nm was used for illumination and set to a 225x225 µm spot size with a measured power of 100 mW. Due to the scan pattern, area of illumination and repetition rate, this measurement results in a ~10 s time point (**Figure S4**).

Structure determination and refinement

The data collected was processed with CrystFEL 0.10.2[59] using peakfinder8 for peak detection and XGANDALF[60] for indexing. For Cristallina data, the optimal settings were determined to be: *--threshold=20 –min-snr=4.0, --min-pix-count=1.*The data was merged and scaled using partialator with the option *-m unity -n 1 --push-res=1.5* and the CCP4 suite[61] used to create MTZ files. Datasets were further treated for anisotropy with the SATARANISO server[62]. For LCLS data, data was merged and scaled using partialator with the option *-m xsphere -n 1* and the CCP4 suite[61] was used to create MTZ files. Dark and light datasets were scaled together during the partialator step and then split using the custom-split option.

To solve the initial models, molecular replacement with the search model 2RH1 was used. To refine the models, iterative cycles of phenix.refine[63] and manual rebuilding in coot were done. Xtrapol8[64] was used to calculate q-weighted difference maps and q-weighted extrapolated maps with standard settings. For LCLS, fewer crystals patterns were collected than for the Cristallina experiment, therefore extrapolation was based on F*calc* of the dark model to improve the quality of the extrapolated map. After visual examination, the activation level was determined to be around 28%. For Cristallina data, the extrapolation was carried from F*obs* from the dark dataset and the activation level was manually determined to be around 22 %. The coordinates of light-activated models were refined against their respective extrapolated maps. After refinement, a mixed model was created for both experiments, containing the appropriate Dark models and Light models according to their determined occupancy. These mix-models were ultimately refined [B-factor and TLS[65]] against their respective light-activated datasets (non-extrapolated). Ligand restraints were generated using eLBOW[66]. The protein model quality was controlled with MolProbity [67].

Molecular Mechanics/Generalized Born Surface Area (MM/GBSA) calculations

Ligand binding energies were estimated using the MM/GBSA method. For this, both receptor ligand complexes (dark and light) were prepared using protein preparation wizard in Schrödinger Maestro[50, 68] using default settings (adding hydrogens, set charges for ionizable groups at pH 7.4 using PropKa and Epik). MM/GBSA calculations were run using Prime on the prepared complexes[50, 69], including an area of 5 Å around the ligand center allowing for protein flexibility to counteract the distorted *cis* ligand conformation. Calculations were performed using Maestro version 2024-3.

Cell Culture

The activity of photoswitchable ligands against β_2AR was evaluated using HEK293 H188 M1 cells, which stably express a cAMP Epac-S^{H188} FRET biosensor[34] and was reported in our previous paper[12b]. We maintained the cell line stably expressing the Epac-SH^{188} cAMP biosensor at 37ºC, 5% (w/v) CO_2 in 4,5 g/L D-glucose Dulbecco's Modified Eagle Medium (DMEM, GIBCO) supplied with 10% heat inactivated FBS (GIBCO) and 1% penicillin-streptomycin (10,000 U/mL, GIBCO). According to the law of mass action, the total amount of spontaneously active

receptors is proportional to their overall concentration in the membrane{Berg, 2018 #827}. Therefore, to increase the number of receptors expressed in the cell membrane and, consequently, the basal activity, HEK293 H188 M1 cells were transiently transfected with β_2AR using lipofectamine. All assays were performed at room temperature. Adherent cells were grown in 150-mm dishes to 75-90% confluence and recovered by rinsing once with PBS (GIBCO), followed by incubation with Trypsin-EDTA (Sigma-Aldrich) for 5 min until detachment of cells was observed. Cells were then centrifugated; in parallel, 10 µL of the single cell suspension were counted using a Neubauer Chamber. The supernatant was carefully removed, and cells were resuspended in DMEM complete medium to obtain a solution at 1.0 x 10^6 cells/mL. 100,000 cells per well were seeded in a transparent 96-well microplate (Thermo Scientific Nunc Microwell) and left at 37ºC with 5% CO_2 for approximately 24h. [34]

Dose-Response Assays

To perform functional assays with azobenzene ligands in HEK293 H188 M1 cells we prepared two different plates, one for each light condition. For all assays, both plates were left to incubate with the studied compounds for 1h at room temperature. To induce photoswitching, the “light plate” was exposed to continuous illumination (380 nm) during 15 min using the LED array plate (LEDA Teleopto). Fluorescence values were thereafter measured and, subsequently, the cells were washed three times with assay buffer. Dose-response curves for each compound were obtained using a constant concentration of the agonist cimaterol (10 nM) in the dark and upon illumination. The cAMP EPAC sensor buffer (14 mM NaCl, 50 nM KCl, 10 nM $MgCl_2$, 10 nM $CaCl_2$, 1 mM HEPES pH 7.2, 1.82 mg/mL Glucose,)[34] supplemented with 100 µM IBMX was used as the assay medium in all FRET-based experiments. Fluorescence values were measured using a Tecan Spark M20 multimode microplate reader equipped with the Fluorescence Top Standard Module and defined wavelength settings (excitation filter 430/20 nm and emission filters 485/20 nm and 535/25 nm). FRET ratio was calculated as the relation of the fluorescent donor emission (td^{cp173}V, 485 nm) divided by the acceptor emission (mTurq2Δ, 535 nm). The FRET ratio was normalized to the effect of the buffer (0%) and the maximum response obtained with cimaterol (100%) or buffer with a constant concentration of the agonist cimaterol (10 nM) (100%) and the maximum antagonism obtained with propranolol (0%). External light was applied using the 96-well LED array plate (LEDA Teleopto). Each set of experiments was performed three to five times with each concentration in duplicate or triplicate.

Binding competition experiments

The binding of photoswitchable ligands towards β_2AR was evaluated using HEK293 cells transiently transfected with β_2AR with lipofectamine. To perform binding competition assays a carazolol-based fluorescent ligand Carazolol-KK114 (Car-KK114) which competes with the azobenzene ligands for the same β_2AR binding site was used[33]. To evaluate the binding affinites of photoswitchable ligands we prepared two different plates, one for each light condition. Both plates were co-incubated with 100 nM of Car-KK114 and different concentrations of the studied compounds for 1h at room temperature. Subsequently, the cells were washed three times with assay buffer and fluorescence values were measured. To induce photoswitching, one plate was exposed to continuous illumination at 380 nm during 15 min using the LED array plate (LEDA Teleopto), while another plate was left under dark conditions for 15 min. Fluorescence values were thereafter measured and, subsequently, the cells were washed and incubated with 100 nM of Car-KK114 for 1h at room temperature. Finally, the cells were washed three times with assay buffer and fluorescence values were measured.

Data analysis

All experiments were analyzed using GraphPad Prism 10.4.0 (GraphPad Software, San Diego, CA). Stimulation dose–response data was fitted using the log(agonist) vs response (three parameters) function. Inhibition dose–response data was fitted using the log (antagonist) vs response (three parameters) function. Competitive binding data was fitted using the One site – Fit Ki function.

Synthesis materials and methods

All starting materials were obtained from commercial sources and used without further purification. Anhydrous solvents were obtained from a solvent purification system (*PureSolv-EN™*) and kept under a nitrogen atmosphere. Reactions were monitored by thin layer chromatography (TLC) on silica gel (60F, 0.2 mm, ALUGRAM Sil G/UV254 *Macherey-Nagel*) and visualized with 254 nm UV light. Reactions under microwave irradiation were carried out in a *CEM Discover Focused*™ Microwave reactor. This instrument is constituted by a continuous focused microwave power delivery system with selectable power output (0-300 W). Reactions were performed in 5 mL sealed glass vessels. Temperature of the vessel content was monitored using an IR sensor and the indicated temperature corresponds to the maximal temperature reached during each experiment. Reaction vessels were magnetically

stirred by means of a rotating magnetic plate located below the floor of the microwave cavity. The specified time corresponds to the total irradiation time. Efficient cooling was accomplished by means of pressurized air during the entire experiment. Flash column chromatography was performed using silica gel 60 (*Panreac*, 40-63 µm mesh) or by means of RediSep Silica (*Biotage*) and/or RediSep HP C18 Gold (*Biotage*) columns, automated with Isolera One with UV-Vis detection (*Biotage*). Nuclear Magnetic Resonance (NMR) spectroscopy was performed using a *400 MHz Brüker Avance NEO 400 MHz* spectrometer. Chemical shifts are reported in δ (ppm) relative to the residual non-deuterated solvent signal (CDCl3 δ = 7.26 ppm (^{1}H), δ = 77.16 ppm (^{13}C); DMSO-*d6* δ = 2.50 ppm (^{1}H), δ = 39.51 ppm (^{13}C), CD3OD δ = 3.31 ppm (^{1}H), δ = 49.3 ppm (^{13}C)). The following abbreviations have been used to designate multiplicities: s=singlet, d=doublet, t=triplet, q=quadruplet, qu=quintuplet, h=heptet, m=multiplet, br=broad signal, dd=doublet of doublet, ddd=doublet of doublet of doublet, dddd=doublet of doublet of doublet of doublet, dt=doublet of triplet, qd=quadruplet of doublet. Coupling constants (*J*) are reported in Hz. High-resolution mass spectra (HRMS) and elemental composition were performed on a FIA (Flux Injected Analysis) with Ultrahigh-Performance Liquid Chromatography (UPLC) *Acquity Premier (Waters)* coupled to LCT Premier Select Series Cyclic IMS (*Waters*). Data from mass spectra was analyzed by electrospray ionization in positive mode using MassLynx 4.2 Software (*Waters*). Spectra were scanned between 50 and 1200 Da with values every 0.4 seconds and peaks are reported as *m/z*. Purity of final compounds was determined by High-Performance Liquid Chromatography (HPLC). Analytical HPLC was performed on a *Thermo Ultimate 3000SD* (*Thermo Scientific Dionex*) coupled to a PDA detector and Mass Spectrometer *LTQ XL ESI-ion trap* (*Thermo Scientific)* (HPLC-PDA-MS)) or on a *Waters 2795 Alliance* coupled to a DAD detector (*Agilent 1100*) and an *ESI Quattro Micro* MS detector (*Waters*); HPLC columns used were *ZORBAX Eclipse Plus C18* (4.6x150mm; 3.5µm) and *ZORBAX Extend-C18* (2.1 x 50 mm, 3.5 µm) respectively. HPLC purity was determined using the following binary solvent system: 5% acetonitrile (v/v) in 0.05% formic acid (v/v) for 0.5 minutes, from 5 to 100% acetonitrile (v/v) in 5 minutes, 100% acetonitrile (v/v) for 1.5 minutes, from 100 to 5% acetonitrile (v/v) in 2 minutes and 5% acetonitrile (v/v) for 2 minutes. The flow rate was 0.5 mL/min, column temperature was fixed to 35 ºC and wavelengths from 210-600 nm were registered. Purity determination was performed with Liquid Chromatography (LC) coupled to a photodiode detector (PDA) and a mass spectrometer (MS). Three different equipment with different methods have been used and are described as followed. Waters 2795 Alliance separation module coupled to a diode array detector (Agilent 1100) scanning at a wavelength range of 210-600 nm and an ESI Quattro Micro MS detector (Waters) in positive mode with mass range (m/z) of 150-1500. A column ZORBAX Extend-C18 3.5 µm 2.1x50mm (Agilent) at 35ºC was used with a mixture of A = H_2O + 0.05% formic acid and B = MeCN + 0.05% formic acid as mobile phase and the method as follows: flow 0.5 mL/min, Gradient t = 0.0 min 5% B, t = 0.5 min 5% B, t = 5.5 min 100% B, t = 7.0 min 100% B, t = 8.0 min 5% B, t = 10.0 min 5% B, total runtime: 10 min.

Synthesis of fluorescent ligand Car-KK114

For the binding experiments the fluorescent ligand Car-KK114 previously reported by Mitronova et al. was used[33]. In addition, Car-KK114 was synthesized following the conditions depicted in Scheme 1. 4-hydroxycarbazol (**1**) was alkylated with glycidyl tosylate (**2**) to form 4-(glycidyloxy)carbazole (**3**). Next, the nucleophilic epoxide ring opening with *tert*-butyl N-[3-amino-3-methylbutyl] carbamate (**4**) followed by the amine deprotection gave the carazolol derivative **6**. Subsequently, a PEG linker was introduced followed through an amide formation with the azido activated carboxylic acid **7** and the resulting azide **8** was reduced by hydrogenation to give the carazolol derivative **9** with a terminal primary amino group, which was used to attach of the fluorescent dye KK114 and form the final Car-KK114 (**10**).

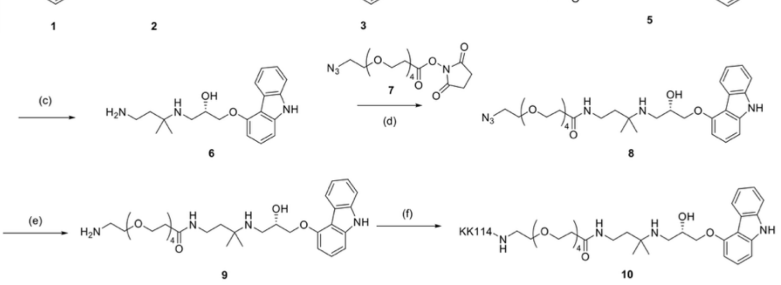

Scheme 1: Synthesis of fluorescent ligand Car-KK114. (a) DMF, rt, ON, 48%; (b) iPrOH, 100 ºC, µW 2h, 76%; (c) TFA, DCM, rt, 6h, 56%; (d) TEA, DMF, 60 ºC, 16h, 56%; (e) H_2, Pd/C, HCl, MeOH, 3 bar, 3h, 16%.; (f) KK114-NHS, TEA, DMSO, rt, 3h, 33%.

***(S)*-4-(oxiran-2-ylmethoxy)-9H-carbazole (3).** 9H-carbazol-4-ol (**1**, 500 mg, 2.73 mmol) and CS_2CO_3 (1.33 g, 4.1 mmol) were dissolved in dry DMF (3 ml) and the reaction mixture was stirred for 10 minutes. *(S)*-oxiran-2-ylmethyl 4-methylbenzenesulfonate (**2**, 934 mg, 4.1 mmol) was added and the reaction mixture was stirred at room temperature overnight. Afterwards, the reaction mixture was concentrated under reduced pressure. Saturated aqueous solution of $NaHCO_3$ and EtOAc were added and the layers were separated. The aqueous layer was extracted with EtOAc twice. The combined organic layers were washed with brine and dried over $MgSO_4$. The crude was purified by automated normal phase *flash* column chromatography (from Hex:EtOAc 85:15 to 25% EtOAc) to give the title compound as a brown solid (653 mg, 48%). ^{1}H NMR (400 MHz, $CDCl_3$) δ 8.34 (d, *J* = 7.8 Hz, 1H), 8.07 (s, 1H), 7.43 – 7.38 (m, 2H), 7.32 (t, *J* = 8.0 Hz, 1H), 7.06 (d, *J* = 8.1 Hz, 1H), 6.66 (d, *J* = 8.0 Hz, 1H), 4.47 (dd, *J* = 11.0, 3.3 Hz, 1H), 4.27 (dd, *J* = 11.0, 5.4 Hz, 1H), 3.60 – 3.52 (m, 1H), 3.00 (t, *J* = 4.5 Hz, 1H), 2.90 (dd, *J* = 4.9, 2.6 Hz, 1H). ^{1}H NMR signals match those reported in literature [70]. HPLC-PDA-MS: RT = 3.64 min, λ_{max} = 242 nm, $[M+H]^+$ = 240.32; purity (254 nm): 100%.

***tert*-butyl (3-amino-3-methylbutyl)carbamate (4).** di-*tert*-butyl dicarbonate (1.18 g, 5.4 mmol) was dissolved in DCM (5 ml). To the ice-cooled mixture, 3-methylbutane-1,3-diamine (1.18 ml, 11.34 mmol) and TEA (1.58 ml, 11.34 mmol) were added and the reaction mixture was stirred at room temperature overnight. Saturated aqueous solution of Na_2CO_3 and DCM were added and the layers were separated. The organic layer was washed with saturated aqueous solution of Na_2CO_3 twice. The combined organic layers were washed with brine and dried over $MgSO_4$. The crude was purified by automated normal phase *flash* column chromatography (MeOH:DCM 90:10%) to give the title compound as a white solid (1 g, 98%). ^{1}H NMR (400 MHz, $CDCl_3$) δ 4.93 (s, 1H), 3.01 (d, J = 6.3 Hz, 2H), 1.45 (s, 9H), 1.10 (s, 6H). ^{1}H NMR signals match those reported in literature[71].

***tert*-butyl *(S)*-(3-((3-((9H-carbazol-4-yl)oxy)-2-hydroxypropyl)amino)-3-methylbutyl)carbamate (5).** 4-(oxiran-2-ylmethoxy)-9H-carbazole (**3**, 300 mg, 1.25 mmol) was dissolved in iPrOH (2 ml). *tert*-butyl (3-amino-3-methylbutyl)carbamate (330 mg, 1.63 mmol) was added and the reaction mixture was stirred at 100 ºC for 2 h at the microwaves. Afterwards, the reaction mixture was concentrated under reduced pressure and the crude was purified by automated reverse-phase *flash* column chromatography (from H_2O:ACN 95:5 + 0.1% HCOOH to 100% ACN + 0.1% HCOOH to give the title compound as a white solid (420 mg, 76%). ^{1}H NMR (400 MHz, $CDCl_3$) δ 8.26 (d, *J* = 7.8 Hz, 1H), 8.23 (s, 1H), 7.43 – 7.35 (m, 2H), 7.32 (t, *J* = 8.0 Hz, 1H), 7.22 (ddd, *J* = 8.1, 6.4, 1.7 Hz, 1H), 7.06 (d, *J* = 8.0 Hz, 1H), 6.67 (d, *J* = 8.0 Hz, 1H), 5.27 (s, 1H), 4.34 – 4.27 (m, 1H), 4.27 – 4.19 (m, 2H), 3.26 – 3.17 (m, 2H), 3.04 – 2.97 (m, 1H), 2.92 – 2.85 (m, 1H), 1.64 – 1.56 (m, 2H), 1.43 (s, 9H), 1.14 (s, 6H). ^{13}C NMR (101 MHz, $CDCl_3$) δ 155.20, 141.10, 138.89, 126.83, 125.16, 123.01, 122.61, 119.78, 112.83, 110.24, 104.05, 101.39, 70.45, 69.34, 52.75, 44.82, 39.96, 36.96, 28.59, 27.17. HPLC-PDA-MS: RT = 3.24 min, λ_{max} = 242 nm, $[M+H]^+$ = 442.48; purity (254 nm): 100%.

***(S)*-1-((9H-carbazol-4-yl)oxy)-3-((4-amino-2-methylbutan-2-yl)amino)propan-2-ol (6).** Trifluoroacetic acid (2.71 ml, 35.2 mmol) was added dropwise to an ice-cooled solution of compound **5** (420 mg, 0.95 mmol) in DCM (2 ml). The reaction mixture was stirred at room temperature for 6 h and was concentrated under reduced pressure to give a crude that was purified by automated reverse-phase *flash* column chromatography (from H_2O:ACN 95:5 + 0.1% HCOOH to 100% ACN + 0.1% HCOOH to give the title compound as a white solid (181 mg, 56%). ^{1}H NMR (400 MHz, MeOD) δ 8.26 (d, *J* = 7.8 Hz, 1H), 7.43 (d, *J* = 8.1 Hz, 1H), 7.37 – 7.32 (m, 1H), 7.30 (t, *J* = 7.5 Hz, 1H), 7.18 – 7.12 (m, 1H), 7.10 (d, *J* = 8.1 Hz, 1H), 6.71 (d, *J* = 7.9 Hz, 1H), 4.50 – 4.43 (m, 1H), 4.40 – 4.34 (m, 1H), 4.30 – 4.24 (m, 1H), 3.53 – 3.46 (m, 1H), 3.35 – 3.26 (m, 1H), 3.12 – 3.03 (m, 2H), 2.15 – 2.07 (m, 2H), 1.44 (s, 3H), 1.43 (s, 3H). ^{13}C NMR (101 MHz, MeOD) δ 155.98, 142.96, 140.76, 127.45, 125.81, 123.64, 123.30, 119.82, 113.50, 111.31, 105.46, 101.65, 70.75, 67.27, 59.51, 45.88, 36.17, 35.99, 23.14, 23.13. HPLC-PDA-MS: RT = 2.31 min, λ_{max} = 242 nm, $[M+H]^+$ = 342.46; purity (254 nm): 100%.

***(S)*-N-(3-((3-((9H-carbazol-4-yl)oxy)-2-hydroxypropyl)amino)-3-methylbutyl)-1-azido-3,6,9,12-tetraoxapentadecan-15-amide (8).** The amine **6** (181 mg, 0.53 mmol) and N_3-PEG_4-$(CH_2)_2$CO-NHS ester (**7**, 206 mg, 0.53 mmol) were dissolved in dry DMF (2 ml), anhydrous TEA was added and the reaction mixture was stirred at 60 ºC overnight. Afterwards, the reaction mixture was concentrated under high vacuum and purified by automated normal phase *flash* column chromatography (from DCM:MeOH 90:10 to 100% DCM) to give the title compound as a white solid (181 mg, 56%). ^{1}H NMR (400 MHz, $CDCl_3$) δ 9.09 (s, 1H), 8.25 (d, *J* = 7.8 Hz, 1H), 7.41 – 7.29 (m, 2H), 7.29 – 7.23 (m, 1H), 7.21 – 7.14 (m, 1H), 7.02 (d, *J* = 8.1 Hz, 1H), 6.60 (d, *J* = 8.0 Hz, 1H), 4.27 – 4.21 (m, 1H), 4.21 – 4.13 (m, 2H), 3.64 – 3.49 (m, 16H), 3.29 – 3.25 (m, 2H), 3.25 – 3.19 (m, 2H), 2.98 – 2.93 (m, 1H), 2.87 – 2.80 (m, 1H), 2.30 (t, *J* = 6.0 Hz, 2H), 1.54 – 1.47 (m, 2H), 1.07 (s, 6H). ^{13}C NMR (101 MHz, $CDCl_3$) δ

171.37, 155.06, 141.20, 139.01, 126.60, 124.90, 122.81, 122.33, 119.34, 112.47, 110.37, 104.12, 100.93, 70.57, 70.54, 70.49, 70.38, 70.28, 70.21, 70.07, 69.90, 69.39, 67.33, 52.44, 50.57, 44.81, 38.89, 37.02, 35.77, 27.15, 27.09. HPLC-PDA-MS: RT = 2.91 min, λ_{max} = 242 nm, $[M+H]^+$ = 615.60; purity (254 nm): 98.5%.

***(S)*-N-(3-((3-((9H-carbazol-4-yl)oxy)-2-hydroxypropyl)amino)-3-methylbutyl)-1-amino-3,6,9,12-tetraoxapentadecan-15-amide (9).** A mixure of azido compound **8** (220 mg, 0.36 mmol), Pd/C (19 mg, 10 % Wt, 0.02 mmol) and HCl (91 µl, 37% in H_2O, 1.11 mmol) were suspended in MeOH (4 ml) and a hydrogen atmosphere of 3 bar was applied for 3 h at room temperature. The reaction mixture was filtered through Celite, washed with MeOH and concentrated under reduced pressure to give a crude that was purified by automated reverse-phase *flash* column chromatography (from H_2O:ACN 95:5 + 0.1% HCOOH to 100% ACN + 0.1% HCOOH to give the title compound as a white solid (35 mg, 16%). ^{1}H NMR (400 MHz, MeOD) δ 8.28 (d, *J* = 7.7 Hz, 1H), 7.46 (d, *J* = 8.1 Hz, 1H), 7.36 (t, *J* = 7.1 Hz, 2H), 7.32 (t, *J* = 8.0 Hz, 1H), 7.17 (t, *J* = 7.4 Hz, 1H), 7.12 (d, *J* = 8.1 Hz, 1H), 6.74 (d, *J* = 7.9 Hz, 1H), 4.56 – 4.47 (m, 1H), 4.44 – 4.37 (m, 1H), 4.34 – 4.27 (m, 1H), 3.73 – 3.65 (m, 4H), 3.64 – 3.49 (m, 14H), 3.38 – 3.33 (m, 2H), 3.09 (t, *J* = 4.9 Hz, 2H), 2.46 (t, *J* = 5.9 Hz, 2H), 2.00 – 1.92 (m, 2H), 1.47 (s, 3H), 1.46 (s, 3H). ^{13}C NMR (101 MHz, MeOD) δ 174.40, 156.00, 142.95, 140.76, 127.50, 125.82, 123.70, 123.32, 119.86, 113.49, 111.36, 105.46, 101.68, 71.29, 71.22, 71.16, 71.12, 70.77, 70.75, 68.09, 67.69, 67.38, 60.11, 45.85, 40.46, 38.25, 37.38, 35.64, 23.79, 23.76. HPLC-PDA-MS: RT = 2.54 min, λ_{max} = 242 nm, $[M+H]^+$ = 589.49; purity (254 nm): 100 %.

Carazolol-KK114 (10). The amine **9** (4.81 mg, 7.69 µmol), a solution of KK114-NHS ester 10,1 mM in DMSO (700 µl, 7.69 µmol) and TEA (23 µl, 11.54 µmol) were dissolved in DMSO (1.0 ml) and the reaction mixture was stirred at room temperature for 3 h. Afterwards, the crude was purified by automated reverse-phase *flash* column chromatography (from H_2O:ACN 85:15 0.1% HCOOH to 45% ACN + 0.1% HCOOH to give the title compound as a blue solid (3.3 mg, 30%). HPLC-PDA-MS: RT = 3.10 min, λ_{max} = 222 nm, $[M+H]^+$ = 1459.82; purity (254 nm): 100%. HRMS (m/z): $[M+H]^{2+}$ calcd for $C_{73}H_{89}F_4N_7O_{16}S_2^{2+}$ 729.7866, found 729.7886.

WILEY-VCH

References

[1] R. Santos, O. Ursu, A. Gaulton, A. P. Bento, R. S. Donadi, C. G. Bologa, A. Karlsson, B. Al-Lazikani, A. Hersey, T. I. Oprea, J. P. Overington, *Nature Reviews Drug Discovery* **2017**, *16*, 19-34.

[2] W. I. Weis, B. K. Kobilka, *Annual Review of Biochemistry, Vol 87* **2018**, *87*, 897-919.

[3] aV. Cherezov, D. M. Rosenbaum, M. A. Hanson, S. G. F. Rasmussen, F. S. Thian, T. S. Kobilka, H. J. Choi, P. Kuhn, W. I. Weis, B. K. Kobilka, R. C. Stevens, *Science* **2007**, *318*, 1258-1265; bS. G. F. Rasmussen, H. J. Choi, D. M. Rosenbaum, T. S. Kobilka, F. S. Thian, P. C. Edwards, M. Burghammer, V. R. P. Ratnala, R. Sanishvili, R. F. Fischetti, G. F. X. Schertler, W. I. Weis, B. K. Kobilka, *Nature* **2007**, *450*, 383-U384; cD. M. Rosenbaum, C. Zhang, J. A. Lyons, R. Holl, D. Aragao, D. H. Arlow, S. G. Rasmussen, H. J. Choi, B. T. Devree, R. K. Sunahara, P. S. Chae, S. H. Gellman, R. O. Dror, D. E. Shaw, W. I. Weis, M. Caffrey, P. Gmeiner, B. K. Kobilka, *Nature* **2011**, *469*, 236-240; dL. Helfinger, C. G. Tate, in *Adrenoceptors* (Eds.: J. G. Baker, M. C. Michel, R. J. Summers), Springer International Publishing, Cham, **2024**, pp. 13-26.

[4] M. M. Papasergi-Scott, G. Pérez-Hernández, H. Batebi, Y. Gao, G. Eskici, A. B. Seven, O. Panova, D. Hilger, M. Casiraghi, F. He, L. Maul, P. Gmeiner, B. K. Kobilka, P. W. Hildebrand, G. Skiniotis, *Nature* **2024**, *629*.

[5] A. Manglik, T. H. Kim, M. Masureel, C. Altenbach, Z. Y. Yang, D. Hilger, M. T. Lerch, T. S. Kobilka, F. S. Thian, W. L. Hubbell, R. S. Prosser, B. K. Kobilka, *Cell* **2015**, *162*, 1431-1431.

[6] R. O. Dror, D. H. Arlow, P. Maragakis, T. J. Mildorf, A. C. Pan, H. F. Xu, D. W. Borhani, D. E. Shaw, *P Natl Acad Sci USA* **2011**, *108*, 18684-18689.

[7] P. Conflitti, E. Lyman, M. S. P. Sansom, P. W. Hildebrand, H. Gutierrez-de-Teran, P. Carloni, T. B. Ansell, S. Yuan, P. Barth, A. S. Robinson, C. G. Tate, D. Gloriam, S. Grzesiek, M. T. Eddy, S. Prosser, V. Limongelli, *Nat Rev Drug Discov* **2025**, *24*, 251-275.

[8] P. Conflitti, E. Lyman, M. S. P. Sansom, P. W. Hildebrand, H. Gutiérrez-de-Terán, P. Carloni, T. B. Ansell, S. G. Yuan, P. Barth, A. S. Robinson, C. G. Tate, D. Gloriam, S. Grzesiek, M. T. Eddy, S. Prosser, V. Limongelli, *Nature Reviews Drug Discovery* **2025**.

[9] aK. Hull, J. Morstein, D. Trauner, *Chem Rev* **2018**, *118*, 10710-10747; bP. Kobauri, F. J. Dekker, W. Szymanski, B. L. Feringa, *Angew Chem Int Edit* **2023**, *62*; cS. Panarello, X. Rovira, A. Llebaria, X. Gómez-Santacana, in *Molecular Photoswitches*, **2022**, pp. 921-944.

[10] aO. Bozovic, B. Jankovic, P. Hamm, *Nature Reviews Chemistry* **2022**, *6*, 112-124; bM. J. Fuchter, *Journal of Medicinal Chemistry* **2020**, *63*, 11436-11447.

[11] A. Sink, H. Gerwe, H. Hübner, V. Boivin-Jahns, J. Fender, K. Lorenz, P. Gmeiner, M. Decker, *Chemistry* **2024**, *30*, e202303506.

[12] aR. Bosma, N. C. Dijon, Y. Zheng, H. Schihada, N. J. Hauwert, S. Shi, M. Arimont, R. Riemens, H. Custers, A. van de Stolpe, H. F. Vischer, M. Wijtmans, N. D. Holliday, D. W. D. Kuster, R. Leurs, *iScience* **2022**, *25*, 104882; bA. Duran Corbera, J. Catena, M. Otero Vinas, A. Llebaria, X. Rovira, *J Med Chem* **2020**.

[13] A. Duran-Corbera, M. Faria, Y. Y. Ma, E. Prats, A. Dias, J. Catena, K. L. Martinez, D. Raldua, A. Llebaria, X. Rovira, *Angew Chem Int Edit* **2022**, *61*.

[14] D. Prischich, A. M. J. Gomila, S. Milla-Navarro, G. Sanguesa, R. Diez-Alarcia, B. Preda, C. Matera, M. Batlle, L. Ramirez, E. Giralt, J. Hernando, E. Guasch, J. J. Meana, P. de la Villa, P. Gorostiza, *Angew Chem Int Ed Engl* **2021**, *60*, 3625-3631.

[15] M. P. Stapleton, *Tex Heart I J* **1997**, *24*, 336-342.

[16] aA. Aquila, M. S. Hunter, R. B. Doak, R. A. Kirian, P. Fromme, T. A. White, J. Andreasson, D. Arnlund, S. Bajt, T. R. Barends, M. Barthelmess, M. J. Bogan, C. Bostedt, H. Bottin, J. D. Bozek, C. Caleman, N. Coppola, J. Davidsson, D. P. DePonte, V. Elser, S. W. Epp, B. Erk, H. Fleckenstein, L. Foucar, M. Frank, R. Fromme, H. Graafsma, I. Grotjohann, L. Gumprecht, J. Hajdu, C. Y. Hampton, A. Hartmann, R. Hartmann, S. Hau-Riege, G. Hauser, H. Hirsemann, P. Holl, J. M. Holton, A. Homke, L. Johansson, N. Kimmel, S. Kassemeyer, F. Krasniqi, K. U. Kuhnel, M. Liang, L. Lomb, E. Malmerberg, S. Marchesini, A. V. Martin, F. R. Maia, M. Messerschmidt, K. Nass, C. Reich, R. Neutze, D. Rolles, B. Rudek, A. Rudenko, I. Schlichting, C. Schmidt, K. E. Schmidt, J. Schulz, M. M. Seibert, R. L. Shoeman, R. Sierra, H. Soltau, D. Starodub, F. Stellato, S. Stern, L. Struder, N. Timneanu, J. Ullrich, X. Wang, G. J. Williams, G. Weidenspointner, U. Weierstall, C. Wunderer, A. Barty, J. C. Spence, H. N. Chapman, *Opt Express* **2012**, *20*, 2706-2716; bJ. Tenboer, S. Basu, N. Zatsepin, K. Pande, D. Milathianaki, M. Frank, M. Hunter, S. Boutet, G. J. Williams, J. E. Koglin, D. Oberthuer, M. Heymann, C. Kupitz, C. Conrad, J. Coe, S. Roy-Chowdhury, U. Weierstall, D. James, D. Wang, T. Grant, A. Barty, O. Yefanov, J. Scales, C. Gati, C. Seuring, V. Srajer, R. Henning, P. Schwander, R. Fromme, A. Ourmazd, K. Moffat, J. J. Van Thor, J. C. Spence, P. Fromme, H. N. Chapman, M. Schmidt, *Science* **2014**, *346*, 1242-1246.

[17] aT. Weinert, P. Skopintsev, D. James, F. Dworkowski, E. Panepucci, D. Kekilli, A. Furrer, S. Brunle, S. Mous, D. Ozerov, P. Nogly, M. T. Wang, J. Standfuss, *Science* **2019**, *365*, 61-+; bG. Khusainov, J. Standfuss, T. Weinert, *Struct Dyn* **2024**, *11*, 020901.

[18] G. Branden, R. Neutze, *Science* **2021**, *373*, 980-+.

[19] T. Gruhl, T. Weinert, M. J. Rodrigues, C. J. Milne, G. Ortolani, K. Nass, E. Nango, S. Sen, P. J. M. Johnson, C. Cirelli, A. Furrer, S. Mous, P. Skopintsev, D. James, F. Dworkowski, P. Bath, D. Kekilli, D. Ozerov, R. Tanaka, H. Glover, C. Bacellar, S. Brunle, C. M. Casadei, A. D. Diethelm, D. Gashi, G. Gotthard, R. Guixa-Gonzalez, Y. Joti, V. Kabanova, G. Knopp, E. Lesca, P. Ma, I. Martiel, J. Muhle, S. Owada, F. Pamula, D. Sarabi, O. Tejero, C. J. Tsai, N. Varma, A. Wach, S. Boutet, K. Tono, P. Nogly, X. Deupi, S. Iwata, R. Neutze, J. Standfuss, G. Schertler, V. Panneels, *Nature* **2023**, *615*, 939-944.

[20] B. Stauch, V. Cherezov, *Annual Review of Biophysics, Vol 47* **2018**, *47*, 377-397.

[21] N. Vaidehi, R. Grisshammer, C. G. Tate, *Trends Pharmacol Sci* **2016**, *37*, 37-46.

[22] A. Ishchenko, B. Stauch, G. W. Han, A. Batyuk, A. Shiriaeva, C. F. Li, N. Zatsepin, U. Weierstall, W. Liu, E. Nango, T. Nakane, R. Tanaka, K. Tono, Y. Joti, S. Iwata, I. Moraes, C. Gati, V. Cherezov, *Iucrj* **2019**, *6*, 1106-1119.

[23] J. A. Ballesteros, H. Weinstein, in *Methods in Neurosciences, Vol. 25* (Ed.: S. C. Sealfon), Academic Press, **1995**, pp. 366-428.

[24] aT. Warne, R. Moukhametzianov, J. G. Baker, R. Nehmé, P. C. Edwards, A. G. W. Leslie, G. F. X. Schertler, C. G. Tate, *Nature* **2011**, *469*, 241-244; bL. Qu, Q. Zhou, Y. Xu, Y. Guo, X. Chen, D. Yao, G. W. Han, Z.-J. Liu, R. C. Stevens, G. Zhong, D. Wu, S. Zhao, *Cell Reports* **2019**, *29*, 2929-2935.e2924.

[25] aC. D. Strader, I. S. Sigal, M. R. Candelore, E. Rands, W. S. Hill, R. A. F. Dixon, *J Biol Chem* **1988**, *263*, 10267-10271; bF. M. Heydenreich, M. Marti-Solano, M. Sandhu, B. K. Kobilka, M. Bouvier, M. M. Babu, *Science* **2023**, *382*, 1378-+.

[26] aP. Nogly, T. Weinert, D. James, S. Carbajo, D. Ozerov, A. Furrer, D. Gashi, V. Borin, P. Skopintsev, K. Jaeger, K. Nass, P. Bath, R. Bosman, J. Koglin, M. Seaberg, T. Lane, D. Kekilli, S. Brunle, T. Tanaka, W. Wu, C. Milne, T. White, A. Barty, U. Weierstall, V. Panneels, E. Nango, S. Iwata, M. Hunter, I. Schapiro, G. Schertler, R. Neutze, J. Standfuss, *Science* **2018**, *361*; bP. Skopintsev, D. Ehrenberg, T. Weinert, D. James, R. K. Kar, P. J. M. Johnson, D. Ozerov, A. Furrer, I. Martiel, F. Dworkowski, K. Nass, G. Knopp, C. Cirelli, C. Arrell, D. Gashi, S. Mous, M. Wranik, T. Gruhl, D. Kekilli, S. Brunle, X. Deupi, G. F. X. Schertler, R. M. Benoit, V. Panneels, P. Nogly, I. Schapiro, C. Milne, J. Heberle, J. Standfuss, *Nature* **2020**.

WILEY-VCH

[27] aT. Nagele, R. Hoche, W. Zinth, J. Wachtveitl, *Chem Phys Lett* **1997**, *272*, 489-495; bI. K. Lednev, T. Q. Ye, R. E. Hester, J. N. Moore, *J Phys Chem-Us* **1996**, *100*, 13338-13341.
[28] aM. Wranik, T. Weinert, C. Slavov, T. Masini, A. Furrer, N. Gaillard, D. Gioia, M. Ferrarotti, D. James, H. Glover, M. Carrillo, D. Kekilli, R. Stipp, P. Skopintsev, S. Brunle, T. Muhlethaler, J. Beale, D. Gashi, K. Nass, D. Ozerov, P. J. M. Johnson, C. Cirelli, C. Bacellar, M. Braun, M. Wang, F. Dworkowski, C. Milne, A. Cavalli, J. Wachtveitl, M. O. Steinmetz, J. Standfuss, *Nat Commun* **2023**, *14*, 903; bT. Weinert, Maximillian Wranik, Jonathan Church, Hans-Peter Seidel, Chavdar Slavov, Tiziana Masini, Daniel James, Hannah Glover, Melissa Carrillo, Demet Kekilli, Robin Stipp, Petr Skopintsev, Steffen Brünle, Natacha Gaillard, Antonia Furrer, Dardan Gashi, Tobias Mühlethaler, John Beale, Karol Nass, Philip J.M. Johnson, Claudio Cirelli, Dmitry Ozerov, Florian Dworkowski, Camila Bacellar, Chris Milne, Michel O. Steinmetz, Josef Wachtveitl, Igor Schapiro, Joerg Standfuss, *Research Square* **2023**.
[29] H. Glover, T. Sassmannshausen, Q. Bertrand, M. Trabuco, C. Slavov, A. Bacchin, F. Andres, Y. Kondo, R. Stipp, M. Wranik, G. Khusainov, M. Carrillo, D. Kekilli, J. Nan, A. Gonzalez, R. B. Cheng, W. Neidhart, T. Weinert, F. Leonarski, F. Dworkowski, M. Kepa, J. Wachtveitl, M. Hennig, J. Standfuss, *Nature Communications* **2024**, *15*.
[30] Y. Kondo, C. Hatton, R. Cheng, M. Trabuco, H. Glover, Q. Bertrand, F. Stierli, H.-P. Seidel, T. Mason, S. Sarma, F. Tellkamp, M. Kepa, F. Dworkowski, P. Mehrabi, M. Hennig, J. Standfuss, *Protein Science* **2025**, *34*, e70104.
[31] aH. C. S. Chan, S. Filipek, S. G. Yuan, *Sci Rep-Uk* **2016**, *6*; bT. Warne, P. C. Edwards, A. G. W. Leslie, C. G. Tate, *Structure* **2012**, *20*, 841-849; cM. Masureel, Y. Z. Zou, L. P. Picard, E. van der Westhuizen, J. P. Mahoney, J. P. G. L. M. Rodrigues, T. J. Mildorf, R. O. Dror, D. E. Shaw, M. Bouvier, E. Pardon, J. Steyaert, R. K. Sunahara, W. I. Weis, C. Zhang, B. K. Kobilka, *Nat Chem Biol* **2018**, *14*, 1059-+.
[32] aD. M. Rosenbaum, C. Zhang, J. A. Lyons, R. Holl, D. Aragao, D. H. Arlow, S. G. F. Rasmussen, H. J. Choi, B. T. DeVree, R. K. Sunahara, P. S. Chae, S. H. Gellman, R. O. Dror, D. E. Shaw, W. I. Weis, M. Caffrey, P. Gmeiner, B. K. Kobilka, *Nature* **2011**, *469*, 236-240; bS. G. F. Rasmussen, B. T. DeVree, Y. Z. Zou, A. C. Kruse, K. Y. Chung, T. S. Kobilka, F. S. Thian, P. S. Chae, E. Pardon, D. Calinski, J. M. Mathiesen, S. T. A. Shah, J. A. Lyons, M. Caffrey, S. H. Gellman, J. Steyaert, G. Skiniotis, W. I. Weis, R. K. Sunahara, B. K. Kobilka, *Nature* **2011**, *477*, 549-U311.
[33] G. Y. Mitronova, G. Lukinavicius, A. N. Butkevich, T. Kohl, V. N. Belov, S. E. Lehnart, S. W. Hell, *Sci Rep-Uk* **2017**, *7*.
[34] J. Klarenbeek, J. Goedhart, A. van Batenburg, D. Groenewald, K. Jalink, *Plos One* **2015**, *10*.
[35] X. Y. Xu, J. Shonberg, J. Kaindl, M. J. Clark, A. Stössel, L. Maul, D. Mayer, H. Hübner, K. Hirata, A. J. Venkatakrishnan, R. O. Dror, B. K. Kobilka, R. K. Sunahara, X. Y. Liu, P. Gmeiner, *Nature Communications* **2023**, *14*.
[36] X. Xu, J. Kaindl, M. J. Clark, H. Hubner, K. Hirata, R. K. Sunahara, P. Gmeiner, B. K. Kobilka, X. Liu, *Cell Res* **2021**, *31*, 569-579.
[37] Y. Z. Cao, S. Shi, S. A. H. Does, C. M. L. Buzink, M. C. Gao, I. J. P. de Esch, H. F. Vischer, M. Wijtmans, R. Leurs, *Journal of Medicinal Chemistry* **2025**.
[38] U. Wirth, E. Neu, D. Provasi, S. Feustel, M. F. Schmidt, H. Hübner, D. Weikert, M. Filizola, B. König, P. Gmeiner, *Angew Chem Int Edit* **2025**, *64*.
[39] Y. X. Han, J. R. D. Dawson, K. R. Demarco, K. C. Rouen, K. Ngo, S. Bekker, V. Yarov-Yarovoy, C. E. Clancy, Y. K. Xiang, S. H. Ahn, I. Vorobyov, *Iscience* **2025**, *28*.
[40] B. I. Gaiser, M. Danielsen, X. Y. Xu, K. R. Jorgensen, P. Fronik, E. Märcher-Rorsted, T. M. Wróbel, X. Y. Liu, J. M. Mathiesen, D. S. Pedersen, *Journal of Medicinal Chemistry* **2024**, *67*, 11053-11068.
[41] A. C. Kruse, B. K. Kobilka, D. Gautam, P. M. Sexton, A. Christopoulos, J. Wess, *Nat Rev Drug Discov* **2014**, *13*, 549-560.
[42] aD. A. Sykes, C. Parry, J. Reilly, P. Wright, R. A. Fairhurst, S. J. Charlton, *Mol Pharmacol* **2014**, *85*, 608-617; bC. J. Dickson, V. Hornak, C. Velez-Vega, D. J. J. McKay, J. Reilly, D. A. Sandham, D. Shaw, R. A. Fairhurs, S. J. Charlton, D. A. Sykes, R. A. Pearlstein, J. S. Duca, *Journal of Medicinal Chemistry* **2016**, *59*, 5780-5789.
[43] H. Nakamichi, T. Okada, *Proc Natl Acad Sci U S A* **2006**, *103*, 12729-12734.
[44] aH. W. Choe, Y. J. Kim, J. H. Park, T. Morizumi, E. F. Pai, N. Krauss, K. P. Hofmann, P. Scheerer, O. P. Ernst, *Nature* **2011**, *471*, 651-U137; bX. Deupi, P. Edwards, A. Singhal, B. Nickle, D. Oprian, G. Schertler, J. Standfuss, *Proc Natl Acad Sci U S A* **2012**, *109*, 119-124.
[45] R. S. Kent, A. De Lean, R. J. Lefkowitz, *Mol Pharmacol* **1980**, *17*, 14-23.
[46] aM. Han, S. O. Smith, T. P. Sakmar, *Biochemistry* **1998**, *37*, 8253-8261; bM. Han, S. W. Lin, M. Minkova, S. O. Smith, T. P. Sakmar, *J Biol Chem* **1996**, *271*, 32337-32342.
[47] E. L. Devine, D. D. Oprian, D. L. Theobald, *P Natl Acad Sci USA* **2013**, *110*, 13351-13355.
[48] D. Weichert, A. C. Kruse, A. Manglik, C. Hiller, C. Zhang, H. Hübner, B. K. Kobilka, P. Gmeiner, *P Natl Acad Sci USA* **2014**, *111*, 10744-10748.
[49] Q. T. Zhou, D. H. Yang, M. Wu, Y. Guo, W. J. Guo, L. Zhong, X. Q. Cai, A. T. Dai, W. J. Jang, E. I. Shakhnovich, Z. J. Liu, R. C. Stevens, N. A. Lambert, M. M. Babu, M. W. Wang, S. W. Zhao, *Elife* **2019**, *8*.
[50] S. Schrödinger Release 2024-3: Maestro, LLC, New York, NY, 2024.
[51] P. Nogly, V. Panneels, G. Nelson, C. Gati, T. Kimura, C. Milne, D. Milathianaki, M. Kubo, W. Wu, C. Conrad, J. Coe, R. Bean, Y. Zhao, P. Bath, R. Dods, R. Harimoorthy, K. R. Beyerlein, J. Rheinberger, D. James, D. DePonte, C. Li, L. Sala, G. J. Williams, M. S. Hunter, J. E. Koglin, P. Berntsen, E. Nango, S. Iwata, H. N. Chapman, P. Fromme, M. Frank, R. Abela, S. Boutet, A. Barty, T. A. White, U. Weierstall, J. Spence, R. Neutze, G. Schertler, J. Standfuss, *Nat Commun* **2016**, *7*, 12314.
[52] R. B. Doak, R. L. Shoeman, A. Gorel, S. Nizinski, T. R. M. Barends, I. Schlichting, *Journal of Applied Crystallography* **2024**, *57*, 1725-1732.
[53] aM. A. Hanson, V. Cherezov, M. T. Griffith, C. B. Roth, V. P. Jaakola, E. Y. Chien, J. Velasquez, P. Kuhn, R. C. Stevens, *Structure* **2008**, *16*, 897-905; bJ. J. Liu, R. Horst, V. Katritch, R. C. Stevens, K. Wüthrich, *Science* **2012**, *335*, 1106-1110.
[54] C. B. Roth, M. A. Hanson, R. C. Stevens, *J Mol Biol* **2008**, *376*, 1305-1319.
[55] A. Ishchenko, B. Stauch, G. W. Han, A. Batyuk, A. Shiriaeva, C. Li, N. Zatsepin, U. Weierstall, W. Liu, E. Nango, T. Nakane, R. Tanaka, K. Tono, Y. Joti, S. Iwata, I. Moraes, C. Gati, V. Cherezov, *IUCrJ* **2019**, *6*, 1106-1119.
[56] R. Andersson, C. Safari, P. Bath, R. Bosman, A. Shilova, P. Dahl, S. Ghosh, A. Dunge, R. Kjeldsen-Jensen, J. Nan, R. L. Shoeman, M. Kloos, R. B. Doak, U. Mueller, R. Neutze, G. Branden, *Acta Crystallogr D Struct Biol* **2019**, *75*, 937-946.
[57] U. Weierstall, D. James, C. Wang, T. A. White, D. Wang, W. Liu, J. C. H. Spence, R. Bruce Doak, G. Nelson, P. Fromme, R. Fromme, I. Grotjohann, C. Kupitz, N. A. Zatsepin, H. Liu, S. Basu, D. Wacker, G. Won Han, V. Katritch, S. Boutet, M. Messerschmidt, G. J. Williams, J. E. Koglin, M. Marvin Seibert, M. Klinker, C. Gati, R. L. Shoeman, A. Barty, H. N. Chapman, R. A. Kirian, K. R. Beyerlein, R. C. Stevens, D. Li, S. T. A. Shah, N. Howe, M. Caffrey, V. Cherezov, *Nature Communications* **2014**, *5*.
[58] R. B. Doak, G. Nass Kovacs, A. Gorel, L. Foucar, T. R. M. Barends, M. L. Grunbein, M. Hilpert, M. Kloos, C. M. Roome, R. L. Shoeman, M. Stricker, K. Tono, D. You, K. Ueda, D. A. Sherrell, R. L. Owen, I. Schlichting, *Acta Crystallogr D Struct Biol* **2018**, *74*, 1000-1007.

WILEY-VCH

[59] aT. A. White, *Acta Crystallogr D Struct Biol* **2019**, *75*, 219-233; bT. A. White, V. Mariani, W. Brehm, O. Yefanov, A. Barty, K. R. Beyerlein, F. Chervinskii, L. Galli, C. Gati, T. Nakane, A. Tolstikova, K. Yamashita, C. H. Yoon, K. Diederichs, H. N. Chapman, *J Appl Crystallogr* **2016**, *49*, 680-689.
[60] Y. Gevorkov, O. Yefanov, A. Barty, T. A. White, V. Mariani, W. Brehm, A. Tolstikova, R.-R. Grigat, H. N. Chapman, *Acta Crystallographica Section A Foundations and Advances* **2019**, *75*, 694-704.
[61] M. D. Winn, C. C. Ballard, K. D. Cowtan, E. J. Dodson, P. Emsley, P. R. Evans, R. M. Keegan, E. B. Krissinel, A. G. W. Leslie, A. McCoy, S. J. McNicholas, G. N. Murshudov, N. S. Pannu, E. A. Potterton, H. R. Powell, R. J. Read, A. Vagin, K. S. Wilson, *Acta Crystallographica Section D Biological Crystallography* **2011**, *67*, 235-242.
[62] I. J. Tickle, Flensburg, C., Keller, P., Paciorek, W., Sharff, A., Vonrhein, C., Bricogne, G. , Cambridge, United Kingdom: Global Phasing Ltd., http://staraniso.globalphasing.org/cgi-bin/staraniso.cgi, **2018**.
[63] P. V. Afonine, R. W. Grosse-Kunstleve, N. Echols, J. J. Headd, N. W. Moriarty, M. Mustyakimov, T. C. Terwilliger, A. Urzhumtsev, P. H. Zwart, P. D. Adams, *Acta Crystallogr D Biol Crystallogr* **2012**, *68*, 352-367.
[64] E. De Zitter, N. Coquelle, P. Oeser, T. R. M. Barends, J. P. Colletier, *Commun Biol* **2022**, *5*, 640.
[65] J. Painter, E. A. Merritt, *Acta Crystallographica Section D-Structural Biology* **2006**, *62*, 439-450.
[66] N. W. Moriarty, R. W. Grosse-Kunstleve, P. D. Adams, *Acta Crystallographica Section D Biological Crystallography* **2009**, *65*, 1074-1080.
[67] V. B. Chen, W. B. Arendall, 3rd, J. J. Headd, D. A. Keedy, R. M. Immormino, G. J. Kapral, L. W. Murray, J. S. Richardson, D. C. Richardson, *Acta Crystallogr D Biol Crystallogr* **2010**, *66*, 12-21.
[68] G. M. Sastry, M. Adzhigirey, T. Day, R. Annabhimoju, W. Sherman, *J Comput Aided Mol Des* **2013**, *27*, 221-234.
[69] aM. P. Jacobson, D. L. Pincus, C. S. Rapp, T. J. Day, B. Honig, D. E. Shaw, R. A. Friesner, *Proteins* **2004**, *55*, 351-367; bM. P. Jacobson, R. A. Friesner, Z. Xiang, B. Honig, *J Mol Biol* **2002**, *320*, 597-608.
[70] M. Stanek, L. P. Picard, M. F. Schmidt, J. M. Kaindl, H. Hübner, M. Bouvier, D. Weikert, P. Gmeiner, *Journal of Medicinal Chemistry* **2019**, *62*, 5111-5131.
[71] C. S. Isfort, T. Kreickmann, T. Pape, R. Fröhlich, F. E. Hahn, *Chem-Eur J* **2007**, *13*, 2344-2357.
WILEY • VCH
